# Power law fitness landscapes and their ability to predict fitness

**DOI:** 10.1038/s41437-018-0143-5

**Published:** 2018-09-06

**Authors:** Diogo Passagem-Santos, Simone Zacarias, Lilia Perfeito

**Affiliations:** Instituto Gulbenkian de Ciencia, Oeiras, Portugal

**Keywords:** Evolutionary theory, Experimental evolution

## Abstract

Whether or not evolution by natural selection is predictable depends on the existence of general patterns shaping the way mutations interact with the genetic background. This interaction, also known as epistasis, has been observed during adaptation (macroscopic epistasis) and in individual mutations (microscopic epistasis). Interestingly, a consistent negative correlation between the fitness effect of beneficial mutations and background fitness (known as diminishing returns epistasis) has been observed across different species and conditions. We tested whether the adaptation pattern of an additional species, *Schizosaccharomyces pombe*, followed the same trend. We used strains that differed by the presence of large karyotype differences and observed the same pattern of fitness convergence. Using these data along with published datasets, we measured the ability of different models to describe adaptation rates. We found that a phenotype-fitness landscape shaped like a power law is able to correctly predict adaptation dynamics in a variety of species and conditions. Furthermore we show that this model can provide a link between the observed macroscopic and microscopic epistasis. It may be very useful in the development of algorithms able to predict the adaptation of microorganisms from measures of the current phenotypes. Overall, our results suggest that even though adaptation quickly slows down, populations adapting to lab conditions may be quite far from a fitness peak.

## Introduction

Evolutionary adaptation is an interplay between stochastic events, such as the appearance of mutations, and quasi-deterministic natural selection. This dual nature leads to an important question: Is adaptation predictable? In theory, a complete understanding of the evolutionary process and all its components would allow us to predict the outcome even of the stochastic components.

Recently, several experiments tested whether or not adaptation is reproducible in a way that allows for a predictive model to be designed (e.g., Long et al. 2015; Wiser et al. 2013; Jerison et al. 2017; Perfeito et al. 2014; Kryazhimskiy et al. 2014; Good et al. 2017). The general conclusion is that the reproducibility of evolution depends on the level of organization that is considered. If we consider the DNA level, there is little reproducibility: the same genotypes adapting to the same conditions will often experience different nucleotide changes. However, as the complexity level increases, the degree of parallelism between different replicate populations also increases. For instance, the fitness increase of replicate populations is highly reproducible. This suggest that, at least for higher-order phenotypes such as fitness, a predictive model can be derived (Kryazhimskiy et al. 2014; Lässig et al. 2017).

One of the least understood components of the adaptive process is the distribution of fitness effects of mutations (DFEM). Classical models of the DFEM consider that the effects of mutations are independent of the background in which they appear. However, there is now plenty of empirical evidence that mutations display some degree of interaction with their background (Lunzer et al. 2005; Trindade et al. 2009; MacLean et al. 2010; Starr and Thornton 2016; de Visser and Krug 2014, for a review). In these studies, specific mutations are introduced in a set of different genetic backgrounds and the change in selection coefficient Δ*s* is measured. This Δ*s* is usually dependent on the background where the mutation is introduced, a phenomenon known as epistasis. Following the terminology introduced by Good and Desai (2015), the term microscopic epistasis refers to the dependence of Δ*s* of a specific mutation on the background where it is introduced. When this dependence is random it is impossible to extrapolate information gathered from one genotype to another. Surprisingly, there is a large body of work with examples where the fitness effects of mutations depends on the fitness of the genotype in a predictable manner (Kryazhimskiy et al. 2014; Perfeito et al. 2014; Woods et al. 2011; Chou et al. 2009). The emerging pattern of negative correlation between Δ*s* and fitness of the background paints a relatively simple view of epistasis which is known as diminishing returns epistasis. This has been observed in different microbial systems, but it must be interpreted with some caveats. For practical reasons it is necessary to select specific mutations and backgrounds to be tested, resulting in a set that might be biased.

Another source of information regarding patterns of epistasis is the study of adaptation experiments. While adapting, populations experience a large number of mutations and their rate of adaptation is highly dependent on the DFEM and its relationship with the available genetic backgrounds. This second form of epistasis, describing the change in properties of the DFEM as a function of the background is known as macroscopic epistasis (Good and Desai 2015). In the majority of scenarios, the rate of adaptation depends negatively on the initial fitness of the background, again a form of diminishing returns epistasis. Although there is no direct link between micro and macroscopic epistasis, it is tantalizing that both follow the same overall trend of diminishing returns. It could be that the same biological mechanisms are behind both, allowing for a single model to explain them.

One of the most prominent classes of models that attempt to tackle the effect of epistasis in adaptation are fitness landscapes (Woods et al. 2011; de Visser and Krug 2014; Bank et al. 2016). Originally proposed by Wright (1932) and Fisher (1930), fitness landscapes describe the mapping between genotype/phenotype and fitness. This map is understood as a fundamental concept in evolutionary biology and plays a crucial role in several theories, such as the evolution of sex (Otto and Lenormand 2002), speciation (Gavrilets 2004), divergence (Chevin et al. 2010), and others. The shape of this map largely influences the predictability of evolution. There have been several attempts to generate fitness landscapes from empirical data. Since the number of experimentally accessible genotypes is small, authors have focused on small regions (see de Visser and Krug 2014, for a review). Most of these empirical landscapes are rugged, but the level of ruggedness varies without any explicit pattern. Additionally, when a larger section of the landscape is explored, the authors find that it is not possible to extrapolate global features from subsets of the data (Bank et al. 2016). The smaller sub-landscapes represent “epistatic hot-spots” that do not necessarily share the overall epistatic pattern. This highlights the risk in extrapolating from small subsets of mutational combinations. It is, however, unclear if a simplified fitness landscape generated with adaptation data, tuned to explain the patterns of macroscopic epistasis, can also be predictive of microscopic epistasis.

**Fig. 1:**
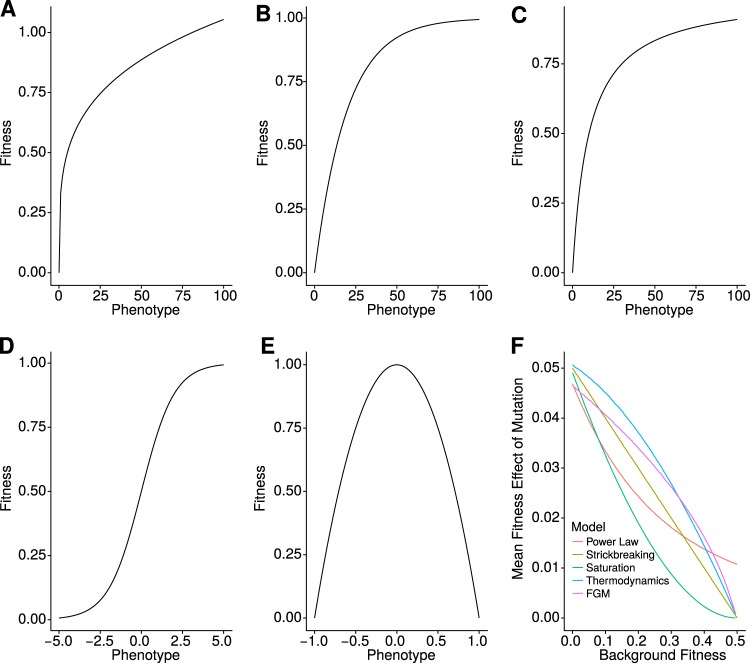
Models of epistasis. **a**–**e** represent the relationship between phenotype and fitness in different models (**a** Power Law, **b** Stickbreaking, **c** Saturation, **d** Thermodynamics, **e** FGM). The equations for each line are in the main text. **f** Mean fitness effect of beneficial mutations as a function of the background fitness for each model

Several different types of fitness landscapes have been proposed, but reviewing all of them is beyond the scope of the present work. Instead, we will focus on two key features related to epistasis. The first is the presence of a nearby fitness optimum, and the second is the curvature of the landscape as the population approaches the optimum. With these two features in mind, we will ask whether one model is able to explain and predict the adaptation of very different populations. We chose five models to test against the data which vary in either curvature or the presence of a maximum. The first is a power law-shaped model without a fitness maximum but with diminishing returns epistasis similar to that proposed by Wiser et al. (2013). We adapted this model to become a phenotype-fitness landscape, directly comparable to the others. As for models with a maximum, we chose four. One of the simplest is the stickbreaking model (Nagel et al. 2012), whereby the fitness effect of beneficial mutations is a linear function of the distance between the current genotype and the maximum. In order to explore non-linear curvatures as the population approaches the optimum, we tested two additional functions. The first is inspired by a Michaellis–Menten type of curve, whereby fitness saturates as phenotypic values increase (Jiang et al. 2013); the second is inspired by thermodynamics and the probability of binding of molecules as a function of affinity (Jacquier et al. 2013). This model produces a sigmoid type of fitness landscape. Our fourth model is Fisher’s Geometrical Model of adaptation (Fisher 1930), which has previously been shown to qualitatively capture the observed patterns of diminishing returns epistasis (Couce and Tenaillon 2015; Blanquart and Bataillon 2016). This model is substantially different from the previous three in that fitness is not a monotonic function of phenotype. Instead there is an intermediate value of phenotype for which fitness is maximum. This leads to, among other properties, sign epistasis. Here we use the extension used in Gros et al. (2009) and Blanquart and Bataillon (2016), which allows different curvatures of the landscape. Figure 1a–e shows the phenotype-fitness function for the five models. These models focus on changes in the fitness effect of mutations. An alternative hypothesis is that the rate or fraction of beneficial mutations changes, rather than its value. One example is the finite sites model (used, e.g., in Zucker 2003), where there is a finite number of beneficial mutations and the pool becomes smaller with increasing number of fixations. We chose not to use these models as they do not predict microepistasis and because there has been little support for the reduction of the fraction of beneficial mutations (Good et al. 2017). Moreover, while our chosen models do not explicitly change the fraction of beneficial mutations, they change the number of mutations that behave as effectively neutral.

We tested the models described above against four published datasets and a new dataset produced by us. The criteria to include the published datasets was the existence of data on macroepistasis (i.e., changes in the general properties of the DFEM during evolution) and data on microepistasis (i.e., description of the fitness effects of mutations in different backgrounds). We also wanted a representation of different species and so we included a new dataset produced by us on the yeast *Schizosacharomyces pombe*. The other datasets include the long-term evolution experiment (LTEE) of the bacterium *Escherichia coli*, and three datasets on the yeast *Saccharomyces cerevisiae*. There are other datasets that explore the adaptation rate of different background (e.g., Perfeito et al. 2014; Schoustra et al. 2016) but these either do not include microepistasis (e.g., Perfeito et al. 2014) or have complex demographic histories such as growth on a structured environment (e.g., Schoustra et al. 2016).

## Methods

### Epistatic models

We tested four different models of epistasis. For each one we fitted the best parameters necessary to explain micro and macroscopic epistasis. Independently of the model, we assume that mutations only affect the growth rate *m*.

#### Power law

Lenski and collaborators proposed a model of epistasis (Wiser et al. 2013) that explains most of the observations on the LTEE. However, since the proposed model is not commutative (Good and Desai 2015), we used a modified version. In the original model, the fitness effect of a mutation was a decreasing function of the number of mutations fixed. This decrease had a characteristic parameter *λ*. As an example, let us take two mutations with selection coefficient *s*_1_ and *s*_2_. Depending on the order of mutations, the fitness of the double mutant will either be *W*_0_ + *s*_1_ + *λs*_2_ or *W*_0_ + *s*_2_ + *λs*_1_. These two are only identical when *s*_2_ = *s*_1_, or *λ* = 1. In order to have a commutative model with the same shape, and to compare it with the other fitness landscapes, we changed the implementation of the power law. Mutations affect a uni-dimensional phenotype *p* linearly and additively. The phenotype is then mapped to growth rate or log-fitness *m* by a power law rule of the form:

1 $$m_m = \alpha (p_m)^k,$$

with *p*_*m*_ being the phenotype value of the genotype, *α* and *k* the model parameters. We assume the phenotypic effects of mutations, Δ*p*, are distributed exponentially with mean *λ*.

#### Stickbreaking

In the stickbreaking model, defined by Nagel et al. (2012), a mutation increases fitness by a specific percentage *α* of the distance to the maximum fitness *m*_max_. The selection coefficient of mutation *i* is given by:

2 $$s_{m_i} = \left( {m_{{\mathrm{max}}} - m_B} \right) \ast \beta _i,$$

where the suffixes *m*_*i*_ and *B* refer to mutation *i* and the background *B* where the mutation appears. Considering that mutations only differ in the value of *β* and that for beneficial mutations 0 < *β* < 1, we assume that overall *β* is distributed as a truncated exponential distribution with rate parameter *γ*. The stickbreaking model can also be turned into a phenotype-fitness landscape by considering that mutations affect a uni-dimensional phenotype linearly and then mapping the fitness effect of each new mutation with the equation above. The growth rate of any mutant *m*_*m*_ with phenotype *p*_*m*_ has the general form:

3 $$m_m = m_{{\mathrm{max}}} - m_{{\mathrm{max}}}(1 - \beta )^p.$$

#### Saturation

Similar to the models described above, the saturation model (Jiang et al. 2013) considers that mutations change phenotype linearly, and growth rate is given by:

4 $$m_m = \frac{{m_{{\mathrm{max}}} \times p_m}}{{p_m + 1}}.$$

#### Thermodynamics

The thermodynamics model is inspired by the probability of protein binding as a function of affinity (Jacquier et al. 2013; Bank et al. 2015) and considers that mutations change phenotype linearly, and the growth rate is given by:

5 $$m_m = \frac{{m_{{\mathrm{max}}}}}{{1 + e^{ - p_m}}}.$$

In all cases, *p*_*m*_ is the phenotype of strain *m*. Mutational changes in phenotype are additive and Δ*p* is drawn from an exponential distribution with parameter *λ*. The fitness of mutant *m* is $W_m = e^{m_m}$.

#### Fisher’s geometric model (FGM)

FGM consists of an *n*-dimensional phenotypic space that is mapped to fitness. Here we consider a simple version of FGM where each mutation is fully pleiotropic and follow Blanquart and Bataillon (2016) where the phenotype to fitness map can have different shapes depending on parameter *Q*, such that

6 $$m_m = m_{{\mathrm{max}}} - \left\| z \right\|^Q,$$

with *m*_max_ being the maximum log-fitness and *z* the Euclidean distance in phenotypic space.

### Experimental evolution of fission yeast

All strains of *Schizosaccharomyces pombe* (fission yeast) were constructed as described in Teresa Avelar et al. (2013) and their genotypes are listed in Table S1. We used eight different strains, four of which contain large chromosome rearrangements produced in the lab through a cre-lox system. In order to construct the rearrangement, loxP sites along with selective resistance markers had to be introduced in specific genes. The different rearrangements differ in the genes where these genetic cassettes were introduced. For each of the four rearrangements, there exists a strain with the wild-type karyotype and the loxP cassettes inserted in the same locus (for full details, see Teresa Avelar et al. 2013). Together, these eight strains consist of a set of different genotypes with different starting fitnesses. Populations of each strain were started from single clones in 96 deep-well plates (maximum volume of 2 ml) containing 500 μL of YES medium (5 g/L yeast extract, 30 g/L glucose, and 225 mg/L of adenine, histidine, leucine, uracil, and lysine) and grown at 32 °C in a shaking incubator (Infors HT Multitron, 230 RPM). Every day cultures were diluted 250-fold, and allowed to grow until saturation (*N* ≈ 10^6^). Each genotype was cultured in 12 independent wells for 160 passages, or 1275 generations. Each genotype was replicated at least 10-fold.

Fitness was measured in competition with an mCherry-expressing reference strain, which has a different genotype from the rest (see strain list). All strains or populations were taken from the −80 °C freezer and grown for 2 days in the same conditions as the evolution experiment. The test populations were then co-cultured with the reference strain under the same conditions. The competition experiment lasted 7 days. Every day the cultures were diluted and distributed to a new deep-well plate and onto a small 96-well plate for measurement. The frequency of each sub-population was tracked by flow cytometry using a BD LSR Fortessa. Analysis of the data was performed using the flowing software (Terho 2013). Selection coefficient *s* was estimated by fitting:

7 $${\mathrm{ln}}\left( {\frac{{f(t)}}{{1 - f(t)}}} \right) = st + {\mathrm{ln}}\left( {\frac{{f(0)}}{{1 - f(0)}}} \right),$$

where *f*(*t*) is the frequency of the unmarked sub-population at time *t*, with time measured in generations of the reference strain. All raw data from this experiment are available in Supplementary File S1 and the median selection coefficient in Supplementary File S2.

### Simulation of adaptation

For each dataset we simulated batch culture experiments with the same overall population dynamics as reported by the authors. The key parameters extracted from each scenario were the initial population size *N*_0_, and the dilution between passages *d* (see Table 1). For each model, we fitted the parameters of the epistatic model under test plus the rate of beneficial *μ*_*b*_ and, when possible, the overall mutation rate *μ*_*n*_. The latter is given by the total number of mutations detected in the population by sequencing.

**Table 1:** Demographic parameters used in simulation

Dataset	Initial population size	Dilution
Fission yeast	10^4^	250
Budding yeast 30	10^5^	1024
Budding yeast 37	10^5^	512
Budding yeast	2 × 10^5^	32
*E. coli*	5 × 10^6^	100

In batch culture experiments populations experience cycles of growth intercalated with instantaneous crashes due to the dilution between passages. Typically, the dynamics of a population in each passage includes an initial phase of slow growth (lag phase), followed by an exponential growth phase, a deceleration phase, and ends with non-growth phase (stationary phase). This dynamics can be complex and adaptation may proceed by changing traits affecting any of these phases. In order to be able to simulate large populations with multiple genotypes, we made a series of simplifications to the population dynamics:

(i) The death rate is null and the observed population growth rate (*m*) is the per capita birth rate. Additionally, at stationary phase *m* = 0, which implies that there is no change in frequency of genotypes at this stage. Populations have no lag or deceleration phase—we assumed that the population is either growing exponentially or arrested at stationary phase. Together with the previous simplification, this means that the *m* we are estimating is an average value over one passage. A common and fixed limiting resource determines the maximum number of cells that can be produced *P* per passage. The carrying capacity of all genotypes is the same and does not change during adaptation. Together with simplification ii, this implies that adaptation can only occur by increasing *m*. Stationary phase is reached when $\mathop {\sum}\nolimits_j^n P_j = P$, with *P*_*j*_ being the number of new individuals produced by *j.* The initial size (*N*(0)) of the population is the same at every passage. Following assumption iii, the size of the population at beginning of passage *i* should be:

8 $$N_i(0) = \frac{{N_{i - 1}(t_{{\mathrm{end}}})}}{d} = \frac{{N_{i - 1}(0) + P}}{d},$$

where *d* is the dilution between passages. Equation (8) can be solved for the equilibrium of *N*_*i*_(0) = *N*_*i*−1_(0) *to get* $N(0) = \frac{P}{{d - 1}}$. Assuming that the observed initial population sizes from Table 1 are already equilibrium values, we can compute the number of new individuals produced per passage as *P* = *N*(0)*(*d* − 1). Our mutation rates *μ* describe the probability of a mutation occurring per cell division. Together with assumption iv, this allows us to compute the total expected number of mutations per passage as *k*_1_ = *μP* = *μN*(0)(*d* − 1). Exponential growth is deterministic. For each individual *j*, the time until cell division is usually considered to be exponentially distributed with mean *m*_*j*_. For each sub-population composed of clonal individuals this dynamics is well approximated by exponential growth of the form

9 $$N_j(t) = N_j(0)e^{m_jt}.$$

As pointed out by others (Wahl and Gerrish 2001), in batch culture experiments much of the stochasticity on the change in frequency of genotypes is due to random sampling between passages rather than stochastic growth. We therefore kept the sampling stochastic but growth deterministic. Supplementary Fig. S9 shows the probability of fixation of different mutations under these conditions. There is only one mutation per cell division.

Given our assumption ii each passage was divided in two different stages: first, we simulated the growth of the population, with the possibility of appearance of new genotypes by mutation, and the growth of these new genotypes. As there is no dynamics in stationary phase (from assumption i), we then simulated the dilution of the population with random sampling of genotypes.

In order to simulate deterministic growth of all *n* genotypes in the population as well as the stochastic appearance of mutations, we started by sampling the number of divisions (*nM*) that will produce a mutated individual from a Poisson distribution with mean *k*_1_. We then randomly selected the *nM* divisions from an uniform distribution between 1 and *P* and found the ordered set *C* = {*c*_1_, *c*_2_,...,*c*_*nM*_}. The time between each mutation *i* and *i* − 1 was obtained by computing how much time Δ*t* it takes for a population of *n* genotypes to produce *c*_*i*_ − *c*_*i*−1_ individuals. Assuming deterministic growth of all genotypes at the time of appearance of *c*_*i*−1_ we found Δ*t* by solving numerically:

10 $$N(t + {\mathrm{\Delta }}t) = N(t)\mathop {\sum}\limits_l^n e^{m_l \ast {\mathrm{\Delta }}t} \Leftrightarrow$$

11 $$N(t_{c_i}) + (c_i - c_{i - 1}) = N\left( {t_{c_i}} \right)\mathop {\sum}\limits_l^n e^{m_l \ast {\mathrm{\Delta }}t},$$

for Δ*t*; $N(t_{c_i})$ was the population size when mutation *i* appeared, *n* the number of different genotypes in the populations, and *m*_*l*_ the growth rate of sub-population *l*. For each mutation we selected the background where it appeared by sampling all genotypes present in the population, weighted by the number of divisions they experience during Δ*t* (weight of genotype *q* is $N_q\left( {t_{c_{i - 1}}} \right) \times \left( {e^{m_q{\mathrm{\Delta }}t} - 1} \right)$). Each mutation produced a new sub-population *r* with size $N_r\left( {t_{c_i}} \right) = 1$ and growth rate given by the models described above.

Several of the analyzed datasets had large population sizes and simulating the previous process became computationally impractical for large mutation rates of beneficial mutations *μ*_*b*_. We used an approximation of a stochastic simulation based on the tau-leaping algorithm (Gillespie 2001). In this case, we allowed the population to evolve without mutation for a certain amount of time, and then introduced the expected number of mutations that happened during that period. For an intermediate level of expected number of mutations per passage 10 < *k*_1_ < 5000 we selected a time step of the order of the mean generation time. This introduces a small error on the timing of mutations, on the order of the variance in growth rate of the population. For *k*_1_ > 5000, the time step was equal to the whole passage, i.e., all mutations appeared at once in the beginning of the growth phase. This last approximation was only necessary for the LTEE dataset with high mutation rate. In that case, the error introduced by the approximation was mitigated by the large number of passages.

At the end of each passage, populations suffered a reduction in size when diluted. This process is expected to be random and therefore we simulated it by sampling the number of individuals from each sub-population from a multivariate hypergeometric distribution with sampling size *N*(0), and the probability of selecting each sub-population *i* equal to its frequency. Again, simulations with a large number of competing sub-populations *k*_2_ were computationally impractical. For intermediate 100 < *k*_2_ < 1000, we approximate the multivariate hypergeometric distribution by a multinomial distribution with the same parameters. For large *k*_2_ > 1000, we approximate this with a Poisson distribution for each sub-population with mean *N*(0)*f*_*i*_, where *f*_*i*_ is the frequency of sub-population *i* at the end of the passage. These two approximations are extensions to the well-known unidimensional approximations of hypergeometric distribution to a binomial distribution (for large populations), or to a Poisson distribution. Stochasticity in the growth phase has been shown to be small compared with sampling with exponential growth and large dilution rates (Wahl and Gerrish 2001). We follow that approximation here. In each simulation we followed the growth rate and number of mutations accumulating in each surviving lineage.

All the simulations were performed using Intel Python 2017 1.3 (Intel 2018).

### Exploring the parameter space

In order to find the sets of parameters for each model that can explain each different dataset we used Approximate Bayesian Computation (ABC). In ABC a random set of parameters is generated from a prior distribution and simulations are performed with these parameters. A set of summary statistics is then calculated for each set *i* of parameters *ss*_*p*_ and the same summary statistics are computed from the observed data *ss*_*o*_. The simulations with *ss*_*p*_ closer to *ss*_*o*_ are selected and the respective parameters represent the posterior distribution.

We selected as summary statistics the normalized median fitness and the number of mutations in evolved clones from each background and time points. Note that we did not use any variance measure to train the models, but we later included it in one of the measures of predictability (cross-correlation analysis as in Zucker 2003). For each parameter set we ran 100 simulations. On the budding yeast datasets, given the large number of backgrounds involved, we clustered the backgrounds by initial fitness using the Freedman–Diaconis algorithm (Freedman and Diaconis 1981). For the LTEE we used only the information up to the initial 10,000 generations.

In order to decrease the number of simulations necessary, we used a two-step ABC strategy. First, we run sequential ABC with the Leonormand implementation on the R package Easy ABC (Jabot et al. 2013). With this method, the prior begins as a bounded uniform distribution and is updated at each step in order to increase the sampling on the parameter regions with best scores. Convergence is achieved when a small fraction of the newly generated points is accepted (5% in our case). Second, we used a post-processing technique implemented in the R package ABC called neural networks (Csilléry et al. 2012; Blum et al. 2010). This strategy trains a neural network to predict the best set of parameters in order to decrease parameter variance (similar to Blanquart and Bataillon 2016). We used the predicted posterior distribution as the prior for a new round of sequential ABC. Finally, we gathered all performed simulations and computed a final posterior distribution for each model in each dataset. We selected the set of parameters with the lowest distance to the data (*d*_min_), and estimated the error in the distance by bootstrap (*ε*). The final posterior distribution was computed by selecting all the parameters that have a distance to the data which is lower than *d*_min_ + 3.9*ε.

The parameters from the sequential ABC were size 1000 and method Leonormand. For ABC neural network we used *numnet* = 1000 and *maxit* = 10,000.

We measured the accuracy of inference of parameters from the adaptation data using cross-validation of our simulated datasets for each model in each dataset, as in Blanquart and Bataillon (2016), with the function *cv4abc* from package abc (Csilléry et al. 2012). For 1000 random simulations (*n*_*cv*_ = 1000) we selected one of our simulations as a pseudo-real target and applied the ABC rejection algorithm on the remaining simulations. We computed the prediction error, defined for each parameter as $\frac{{{\sum} (\tilde \theta _i - \theta _i)^2}}{{n_{cv} \ast V[\theta ]}}$, where $\tilde \theta _i$ is the median of the posterior distribution, *θ*_*i*_ the true value of parameter on simulation *i*, and *V*[*θ*] the variance of the prior. A value close to 0 reflects perfect inference, while a value of 1 indicates that no inference can be made.

### Normalization of fitness data

The definition of fitness is not the same between the different studies from which we selected our data. In order to be able to compare between datasets we defined fitness of a strain *i* as its mean growth rate over one passage *m*_*i*_. For each dataset we also consider the unit of time as the mean generation time of the reference *r*, so *m*_*r*_ = ln2. The selection coefficient of a mutation *i* is then:

12 $$s_i = m_i - m_r.$$

Below we describe the precise transformation between the fitness we computed and the one reported by the authors. For each dataset, with *s*_*n*_ as our normalized *s* and *s*_*o*_ as the original definition from the authors:

- *E*. *coli*: *s*_*n*_ = (*s*_*o*_ − 1) ln 2.
- Budding yeast: $s_n = \frac{{{\mathrm{ln}}\frac{{f_m(t)}}{{f_r(t)}} - {\mathrm{ln}}\frac{{f_m(0)}}{{f_r(0)}}}}{{G_p + {\mathrm{log}}_2\frac{{f_r(t)}}{{f_r(0)}}}}$, where *f*_*m*_ and *f*_*r*_ represent the frequency of the mutant and the reference, respectively, and *G*_*p*_ represents the generations of the population.
- Fission yeast: We used the same equation as for budding yeast, but since we had multiple data points we estimated fitness using linear regression.

### Statistical analysis

All statistic analysis were done with R 3.4.2 (R Core Team 2015).

We performed model comparison using two measures from the ABC methodology and implemented on the R package abc (Csilléry et al. 2012) as *gfit* and *postpr*. The first, posterior predictive checks, measures the goodness of fit (GoF) of the model to the data. In summary, for each model in each dataset we selected one random simulation as pseudo-real and computed the median of the distance of the closest 1% other simulations. We repeated this procedure 1000 times to generate a null distribution of distances. We then compared the distance between the best 1% simulations and the observed data, and computed an empirical *p*-value, that we corrected for multiple testing using Holm’s method (Holm 1979). The second measure, model selection, pools all simulations from all models and parameters and picks the top 10% closest to the experimental summary statistics. A neural network is then trained to assign a probability to each model. This measure has been shown to be less sensitive to biases in the priors (Csilléry et al. 2012).

In order to compare the observed evolutionary histories with the model outcome and estimate each model’s predictability, we used two correlation methods. We started by simulating the expected evolutionary history (fitness and number of fixed mutations per background per timepoint) under each model in each experimental condition using the inferred posterior distribution of parameters. We then calculated the coefficient of correlation (*r*^2^) and the cross-correlation score (Zucker 2003). The *r*^2^ indicates how much of the variance in the observed adaptation rate is explained by the model. The cross-correlation score uses all biological replicates and estimates Spearman’s correlation between their distribution and the distribution of outcomes from the simulations. Unlike the *r*^2^, this measure takes into account the variance produced by the stochasticity of the evolutionary process. We used bootstrap of the expected evolutionary history to compute a distribution of both *r*^2^ and cross-correlation. Note that the summary statistics we used for the ABC fitting are the same as the experimental measures of the evolutionary trajectories (fitness and number of mutations).

We also used the R packages matrixStats (Bengtsson 2017), boot (Davison 2006; Canty and Ripley 2012), tmvtnorm (Wilhelm 2015), corpcor (Schäfer et al. 2014), Hmisc (Harrell 2018), MBESS (Kelley 2018), and truncnorm (Trautmann et al. 2014).

### Test models in new data

**Fig. 2:**
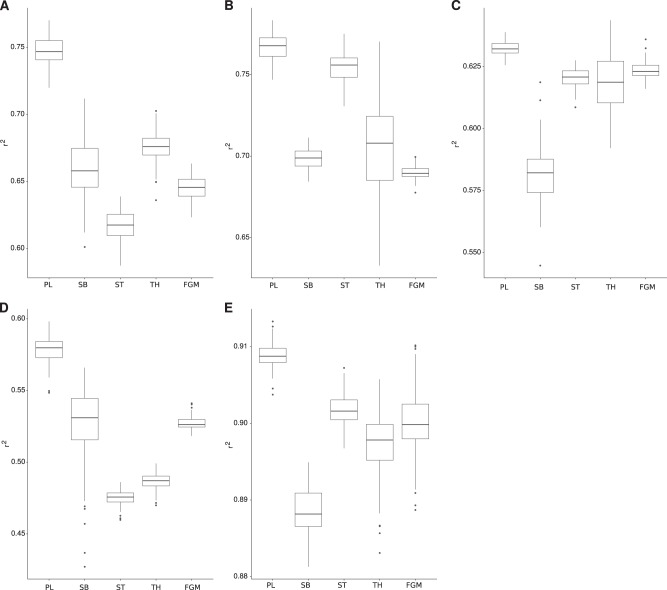
Model comparison. Comparison of the *r*^2^ using simulations with the best set of parameters for each model and dataset, selected using ABC. The model names are abbreviated (Power Law—PL, Stickbreaking—SB, Saturation—ST, Thermodynamics—TH, FGM). **a** Fission yeast, **b** budding yeast 30, **c** budding yeast 37, **d** budding yeast, **e** *E. coli*, and used to compute the correlation coefficient *r*^2^

In order to test influence of the amount of data in the observed *r*^2^ (Fig. 2a), we selected the posterior distribution with the same methodology described above but using only the information available up to some generations. Specifically, given that the fission yeast experiment was performed for 1200 generations a fraction of 50% represent that only data up to 600 generations was used to select the posterior distribution of parameters. Then we used the median of the complete set of simulations from these parameters to calculate the *r*^2^ for the entire dataset.

In one of the budding yeast datasets, the authors measured fitness at two different timepoints. As above, we used only the data from the first timepoint to select the posterior distribution of parameters. We ran simulations using this posterior distribution and computed the median predicted fitness for each background at 500 generations (Fig. 2b).

We performed a similar experiment with the LTEE. Using the posterior distribution of parameters computed with the initial 10,000 generations we performed simulations for the full time of the experiment (50,000 generations). We also show the fit of a linear model to the data of 20,000–50,000 generations (as proposed by Good and Desai 2015).

### Microepistasis

**Fig. 3:**
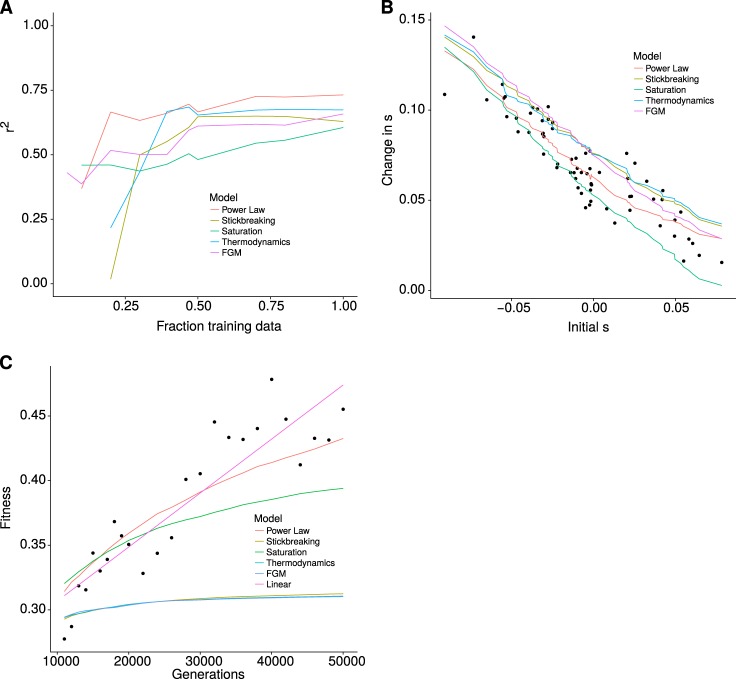
Model robustness to partial data. **a** The correlation coefficient *r*^2^ was computed for the Fission yeast dataset using as training set different fractions of the data. **b** Using only the data for the first 250 generations of the Budding yeast dataset fitness was predicted for 500 generations. The coefficient of correlation *r*^2^ as computed between the predicted and the observed fitness at 500 generations for each model (Power Law *r*^2^ = 0.48, Stickbreaking *r*^2^ = 0.03, Saturation *r*^2^ = 0.18, Thermodynamics *r*^2^ = −0.02, and FGM *r*^2^ = 0.05). **c** Using the informations on the initial 10,000 generations the next 40,000 generations of the LTEE were predicted. The coefficient of correlation *r*^2^ as computed between the predicted and the observed fitness for the last 40,000 generations for each model (linear *r*^2^ = 0.82, Power Law *r*^2^ = 0.78, Stickbreaking *r*^2^ = −1.6, Saturation *r*^2^ = 0.48, Thermodynamics *r*^2^ = −1.7, and FGM *r*^2^ = −1.8)

From both Kryazhimskiy et al. (2014) and Khan et al. (2011), we retrieved the data concerning the effect of mutations in different backgrounds. We removed all mutations that showed either no epistasis (one in each dataset) or positive epistasis (from the LTEE dataset). For the power law model, we considered the *α* and *k* estimated from the respective adaptation dataset and fitted a Δ*p* for each mutation. For the stickbreaking, saturation, and thermodynamics models, we considered the maximum fitness estimated from the adaptation data and also fitted a Δ*p* for each mutation. The distributions displayed in Fig. 3a and b result from sampling from the posterior distribution of parameters for each model, while Fig. 3c and d showed the result from the best set of parameters for microepistasis for the power law model.

## Results

### General model of epistasis

The ubiquitous observation of diminishing returns epistasis in different systems (Couce and Tenaillon 2015) suggests a general model for the selection coefficient *s* of new mutations and the DFEM. Here, we tested two important properties of fitness landscapes that may help us understand diminishing returns epistasis and predict adaptation rates. The first property we tested is whether the observed diminishing returns epistasis of beneficial mutations requires the proximity of a fitness optimum.

We began by choosing a model without a maximum, but with the potential to create diminishing returns epistasis. We chose a power law model, inspired on the observations from the LTEE (Lenski et al. 1991) that selection coefficient and time are related by a power law (Wiser et al. 2013). This model does not have a maximum, i.e., fitness tends to infinity as beneficial mutations accumulate. We modified the empirical model used by the authors in order to make it commutative (i.e., so that the order of mutations does not matter) and with an explicit phenotype-fitness landscape associated. Our new model assumes that mutations change a general phenotype *p* linearly and that this phenotype in turn can be mapped to growth rate *m* by a power law relationship of the type:

13 $$m = \alpha p^k,$$

with *α* being a scale parameter and *k* modulating the degree of epistasis. The selection coefficient *s* is the difference between growth rates (*s* = *m*_mutant_ − *m*_background_). As long as 0 < *k* < 1 the model predicts diminishing returns epistasis, i.e., the selection coefficient of a mutation that changes the phenotype by Δ*p* is inversely correlated with the background fitness (Fig. 1a). In order to simulate the adaptive process, we considered that each mutation has a specific Δ*p*, and that these values are additive and distributed exponentially with mean *λ*. Consistent with diminishing returns epistasis, the power law model predicts a slowdown in the adaptation rate as the population increases in selection coefficient (Fig. 1f, red line).

The second property of fitness landscapes that we tested was the curvature of the phenotype-fitness map when there is a maximum fitness. For that, we tested four models with different properties. The first was the stickbreaking model from Nagel and colleagues (2012). This model assumes that a maximum growth rate *m*_max_ exists and that each individual mutation increases the selection coefficient by a fraction *β* of the distance to the maximum, such that:

14 $$m_{{\mathrm{mutant}}} = m_{{\mathrm{max}}} - m_{{\mathrm{max}}} \ast (1 - \beta )^p.$$

The second model with a maximum that we tested was the saturation model:

15 $$m_{{\mathrm{mutant}}} = m_{{\mathrm{max}}}\frac{p}{{p + 1}}.$$

This model is inspired by a Michaelis–Menten type of relationship between phenotype and fitness. In the biochemical model, the speed of a reaction increases with the concentration of substrate, but it decelerates for high substrate and has an asymptote. Here the substrate is the phenotype *p* and the speed is fitness. Just like in the stickbreaking model, the landscape is concave (Fig. 1b, c) but the change in mean selection coefficient with background is different (Fig. 1f, green and blue lines).

A different type of model is inspired by thermodynamics (Jacquier et al. 2013; Bank et al. 2015), which we formulate as:

16 $$m_{{\mathrm{mutant}}} = \frac{{m_{{\mathrm{max}}}}}{{1 + e^{ - p}}},$$

where fitness is at first a convex (i.e., accelerating) function of phenotype that then becomes concave (Fig. 1d). This model has a characteristic type of microepistasis (Fig. 1f, pink line).

Lastly, we tested FGM, which in Couce and Tenaillon (2015) was shown to best describe the experimental patterns qualitatively. FGM can have different formulations with multiple parameters. Here we decided to use one of the simplest versions in order to compare with the other models which only have three or four parameters. In this version of FGM, mutations are fully pleiotropic and can affect *n* phenotypes randomly. Fitness is a multivariate function of these phenotypes with parameter *Q* describing its shape, as described in Blanquart and Bataillon (2016). Specifically,

17 $$m_{{\mathrm{mutant}}} = m_{{\mathrm{\max}}} - \left\| z \right\|^Q,$$

with ||*z*|| as the Euclidean distance of the mutant phenotypes to the optimal phenotypic values. One of the biggest differences between this model and the previous is that the optimum fitness is not given by the maximum phenotype, but rather by an intermediate value (Fig. 1e). The shape of microepistasis is similar to the thermodynamics model (Fig. 1f, green line).

In all models, only one parameter is specific of each mutation. The rest are specific of the landscape. Hence we will be able to fit the model to data on adaptation rate (macroepistasis) and use it to predict the fitness effects of single mutations with only one free parameter. As Fig. 1f shows, these models make different predictions on the average shape of microepistasis. Supplementary Fig. S1 shows the full distribution of beneficial mutations for each model in two backgrounds.

### Power law model as the general model

The models defined above produce predictions for both micro and macroscopic epistasis. One way to test for microscopic epistasis is to measure the effects of the same mutation in different backgrounds. Although this strategy allows for a thorough description of the fitness landscape for a specific mutation, it requires an initial selection of the mutations and backgrounds to be tested, which can lead to a bias on the observed behavior. Adaptation experiments, on the other hand, are biased in a different way in that only strongly beneficial mutations are sampled. We selected a set of experiments performed with different conditions, measuring adaptation on prokaryotes and eukaryotes. When possible, we tested whether patterns of macroepistasis can explain microepistasis.

In all cases, we used an ABC approach to find the best parameters that explain the observed fitness changes over time. We simulated the evolution experiments, including the demographic dynamics described by the authors of the datasets. For all models, the best parameters are shown in Table 2 and Supplementary Tables S2–6. We measured the accuracy of parameter inference under this methodology with cross-validation and found that, for the majority of scenarios, we can find the true parameters underlying the adaptations dynamics (Supplementary File S3).

**Table 2:** Best parameters for power law model

Dataset	Rate beneficial mutations	Rate neutral mutation	*α*	*k*	*λ*
*E. coli*	8.5*e* − 05	1.1*e* − 03	0.32	0.13	66
Budding yeast	6.0*e* − 05	1.9*e* − 02	0.40	0.20	1.2
Budding yeast 30	3.0*e* − 04	1.0*e* − 03	0.26	0.27	1.2
Budding yeast 37	2.0*e* − 04	2.0*e* − 03	0.13	0.41	1.9
Fission yeast	2.3*e* − 05		0.31	0.13	57

The ABC framework provides two standard methods to compare different models. The posterior predict check (also know as GoF test) measures the GoF of the model to the data by testing if the model can recapitulate the observed summary statistics. The method generates an empirical *p*-value (Supplementary Table S7) that can be used to reject models. In the second method, model selection, a neural network is trained with the best simulations across all the models for the same dataset and the probability of each model is estimated (Supplementary Fig. S3). We applied both of these methods to all models in all datasets and found that there was little power to reject any of the models with the GoF empirical *p*-values. However, the model selection method indicated that the power law had the highest model probability in all dataset except one, budding yeast at 37 °C. In this case, the power law and the saturation model were the most top picks with similar probabilities (Fig. S3C).

**Fig. 4:**
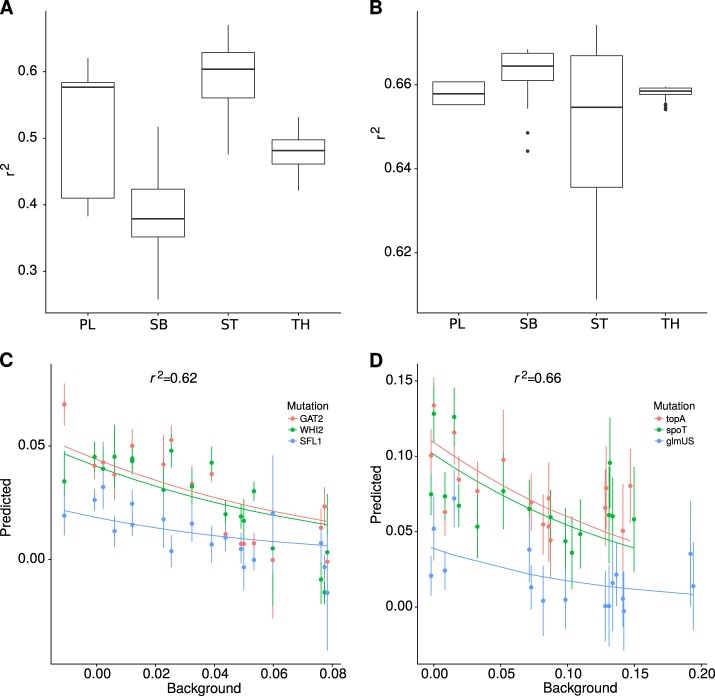
Microepistasis. The model names are abbreviated (Power Law—PL, Stickbreaking—SB, Saturation—ST, Thermodynamics—TH, FGM). **a** The correlation coefficient for microepistasis on the Budding yeast dataset was computed using the general parameters inferred from the adaptation data for each model except FGM. **b** The correlation coefficient for microepistasis on the LTEE dataset was computed using the general parameters inferred from the adaptation data for each model except FGM. **c**, **d** Simulations using the best set of parameters for microepistasis obtained for the power law model using the information from adaptation on the Budding yeast or LTEE data, respectively. Poits represent the experimental data and lines the model prediction

Since our main goal was to assess the predictive potential of these models, we measured the ability of each one to recapitulate the evolutionary history present in the data. For this purpose we computed the coefficient of correlation, *r*^2^ (Fig. 4), and estimated the cross-correlation (Zucker 2003) (Supplementary Fig. S2) between the output of the model and the experimental data. While the *r*^2^ measures the amount of variance in the observed data explained by the model, the cross-correlation also evaluates the ability of the model to recapitulate the stochasticity in the evolutionary process (see Methods). These measures may also help us choose between models since the summary statistics we used for the ABC are the same as the authors reported for the evolutionary trajectories (fitness measures and number of acquired mutations).

All of the measures, both from model comparison and from model predictability, have strengths and weaknesses. Therefore, we searched for robust global patterns across datasets and measures.

The first dataset we looked at was using the fission yeast *S. pombe*. In this experiment, we propagated eight strains which differ by the presence of cre-lox cassettes at different loci and by the presence of large chromosome rearrangements (Teresa Avelar et al. 2013, Methods and Table S1). Replicate populations were evolved for about 1000 generations and fitness was measured at regular intervals (see Supplementary Files S2 and S3). As shown in other organisms, the adaptation rate was inversely correlated with initial fitness, with the less fit strains showing the highest fitness increase. We fitted all five models to this data (Supplementary Fig. S4). While we were unable to reject any of the models (*p*-value ≥0.05, Supplementary Table S7, first column), the power law was the model with the highest probability (Supplementary Fig. S3 panel A). This result was also observed for both predictability measures (Fig. 4 and Supplementary Fig. S2, both panel A), with the power law model having better predictability, followed by stickbreaking and the thermodynamics model.

We repeated the same analysis for the dataset described in Kryazhimskiy et al. (2014), where 66 strains of the budding yeast *S. cerevisisae* were propagated for about 500 generations and their competitive fitness measured at two timepoints. For this and the following datasets we have information on the average number of mutations acquired per line and we used that information for the fit and to infer the overall mutation rate (see Table 2 and Supplementary Tables S2–S6). As above, no model was rejected (*p*-value ≥0.05, Supplementary Table S7, second column) and the power law had the highest model probability (Supplementary Fig. S3 panel B). As for the model predictability, while power law displayed the higher *r*^2^ (Fig. 4b) the best cross-correlation score was from the thermodynamics model, closely followed by the power law (Supplementary Fig. S2 panel B).

Next, we used two additional datasets on budding yeast produced by Jerison et al. (2017). In this case, 230 different strains were adapted for 500 generations in two different temperatures: 30 and 37 °C. For both datasets the model comparison results were similar to the ones described above, with no model being rejected (Supplementary Table S7, third and fourth column). The power law was the model with the higher probability (Supplementary Fig. S3 panels C and D), although very similar to the Saturation model on the 30° dataset. The power law was also the model with the higher predictability measured by *r*^2^ (Fig. 4 panels C and D) and by cross-correlation on the 37° dataset (Supplementary Fig. S2 panel D). Similarly to the previous dataset, for the 30° data the Saturation model had the higher cross-correlation score (Supplementary Fig. S2 panel C), closely followed by the power law model. It is interesting to note that the previous dataset was also grown at 30°, even though the genetic backgrounds are very different.

Finally, we took advantage of a large dataset from the Long-Term Experiment in *E. coli*. Twelve populations of this bacterium were started from the same genotype about 30 years ago and have been evolving to this day. We took the first 10,000 generations of adaptation and fitted them to the models as described above. As with the yeast, no model could be rejected (Supplementary Table S7, fifth column) and the power law was the model with higher model probability (Supplementary Fig. S3 panels E) and predictability (Fig. 4 and Supplementary Fig. S2, both panel E).

We find that the power law is a very strong candidate for a general model, able to explain macroepistasis across prokaryotes and eukaryotes. In Table 2, we show for each dataset the parameters that best fit each of the datasets (Supplementary Tables S2–S6 show the parameters for the other models). The estimated mutation rates are within what was previously described for these organisms. The parameters of the power law differ between datasets but we do not have a clear a priori expectation for their values. In the discussion section we speculate about their biological significance.

### Model robustness to partial data

In order to assess the predictive power of each model, we first estimated how much predictability changed with the fraction of data used. We focused on predicting future fitness only, i.e., we used data from early time points to predict subsequent fitness. In all cases we did not fit any parameter to the datasets to be predicted. We took our fission yeast data and used only an initial fraction of the timepoints to predict the following fitness. Figure 2a shows how the *r*^2^ of each model changes with the fraction of data used in training. It shows that information on the first 500 generations of the experiment was sufficient to achieve the maximum levels of *r*^2^ for all models. The power law performs best irrespective of the amount of training data used. In the yeast dataset with 66 yeast genotypes adapting to standard laboratory conditions (Kryazhimskiy et al. 2014), the authors report fitness for two data points, generation 250 and generation 500. We used the data from generation 250 to predict fitness at generation 500. The results are shown in Fig. 2b. All models capture the qualitative dynamics of the experiment. However, the power law shows the best fit with an *r*^2^ of 0.48. We also tested the ability of the models to predict fitness in the later generations of the LTEE. We used only the data from the first 10,000 generations to train the data and try to predict fitness until generation 50,000. Of all the five models we described before, the power law is the one that best fits the data. It has recently been proposed that this part of the LTEE has a different dynamics in the last generations which in fact shows no signs of epistasis (Good and Desai 2015). To test for this, we fitted a straight line to the LTEE in this interval. The *r*^2^ is slightly higher but similar to that of the power law.

### Prediction of microepistasis

**Fig. 5:**
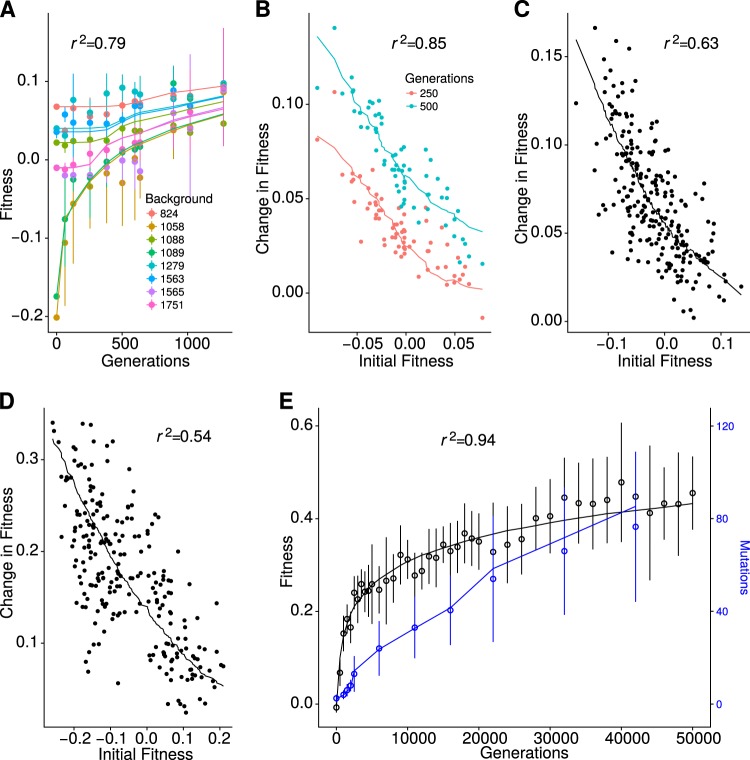
Power law. The predictions obtained from the best set of parameters for the power law model. **a** Fission yeast, **b** budding yeast, **c** budding yeast 30, **d** budding yeast 37, **e** *E. coli*; points represent the experimental data and lines represent the results of simulations, smoothed over multiple replicates

Another independent measure of predictability in these datasets is microepistasis. Both the LTEE (from Khan et al. 2011) and the yeast dataset (from Kryazhimskiy et al. 2014) have data on the fitness effect of single mutations across backgrounds. In both cases, we focused on mutations that show diminishing returns epistasis and no sign epistasis. Exceptions to diminishing return epistasis certainly exist, but we do not intend to explain them here. Rather we are focusing on the more common pattern. Hence we removed two mutations from the LTEE that showed synergistic epistasis or no epistasis and one mutation from Kryazhimskiy et al. (2014) that had no effect on fitness. For each model except FGM, we used all the parameter values estimated from the fitness trajectories described above. For each mutation, only one parameter was fitted: the fitness effect on phenotype, Δ*p*. In order to fit FGM to microepistasis, we would need to fit for each background and mutation the phenotypic value in each dimension *n* of the landscape. The number of free parameters would be very large and it would make the comparison with the other models difficult to interpret. Hence we opted not to use FGM to predict microepistasis. All models are able to capture the trend of diminishing returns epistasis with similar *r*^2^ (Fig. 5a, b) but no clear pattern of one model being better than the other. This result clearly demonstrates that we can make a direct link between macro and microepistasis, with the prediction for the power law model in Fig. 2c and d.

## Discussion

The seemingly pervasive diminishing returns epistasis, whereby the fitness effect of new mutations depends on initial fitness, suggests that a general model of adaptation can be derived. In this work we selected five models and tested their ability to describe and predict patterns in two different experiments: a bound-less model, the power law; three bounded models with different curvatures; and Fisher’s geometrical model. We observe that the power law fits well experimental data from three species in different environments. Although standard goodness-of-fit methods do not allow us to reject any of the models, we observe consistently that the power law has the best scores. Moreover, when we use correlation measures such as *r*^2^, the power law again is the highest ranking model across datasets. There is a caveat that the power law has one additional parameter when comparing with the first three bounded models. It is unlikely that this is the reason behind the best fit because the other three models have sufficient differences in curvature that one would expect that at least one of them would provide a better fit. In addition, the predictive capacity of the power law remains when we test it against new data (Figs. 2, 3). This shows that, even if it were not the best model, it is the more useful. Figure 5 shows for all datasets the experimental data along with the power law prediction with the best fitted parameters.

Previous work conducted a similar unification of data from different sources (Couce and Tenaillon 2015). There, the authors tested four models: a model similar to the power law where the effect of mutations changes exponentially with initial fitness; a model where the number of mutation decreases as an exponential function of initial fitness; the finite sites model; and the FGM. The authors found that FGM was the most robust model in qualitatively describing diminishing returns epistasis. Here, we expand on that analysis by including models with different curvatures toward the maximum and quantitatively analyzing how well each model explains each dataset. We chose five models based on whether or not they had a maximum fitness and on the curvature of the phenotype-fitness map (Fig. 1a–e). Importantly, they all make a different range of predictions on microepistasis (Fig. 1f). The datasets we chose include three different species. We found that the power law model has a better fit to the observed data for all datasets in nearly all measures (Fig. 4, S2).

These five models were chosen based on their simplicity or the fact that they were previously shown to have some power to describe adaptation experiments (Nagel et al. 2012; Wiser et al. 2013; Couce and Tenaillon 2015). They are not explicitly describing any known biological mechanism which makes their interpretation difficult. However, by analyzing the power law model and the behavior of micro and macroscopic epistasis as we change the parameters values it is possible to assign some meaning to each parameter:

- *ɑ*-scale parameter: All *s* are relative to the reference used in the study and *α* defines what is the fitness value of one unit of *p* in units of generation time of the reference. This value should be specific to each experimental condition.
- *k*-epistatic parameter: This parameter defines the strength of epistasis in the system. With *k* = 0 mutations cannot change fitness (a limit scenario) and as it increases, the model predicts less epistasis until *k* = 1 where mutations are non-epistatic. This parameter represents a summary of some underlying complex fitness landscape and thus should be shared between micro and macroscopic epistasis.
- *λ*-mean change in phenotype: This parameter defines how fast the underlying phenotype changes with mutations. The phenotype *p* represents a projection of the fitness-related phenotypes to one dimension and represents the mean change in this effective phenotypic dimension.

In order to simulate adaptation under the different models, we had to make a number of simplifications. Two of them in particular have consequences on how our results are interpreted. The first one was that the growth between passages was described by a single parameter *m*. One way to give biological meaning the power law landscape will be do dissect how the different phases of growth contribute to fitness. The second simplification was that we only incorporated genetic drift at the sampling between passages rather than during cell division. We chose to simulate explicitly the exponential growth of populations instead of using discrete generations with an appropriate effective population size. This allowed us to include any impact of severe, periodic bottlenecks from the experimental settings as seen in Wahl et al. (2002). However, we had to make the simplification that growth is deterministic and all the stochasticity associated with drift originates from the random sampling between passages. Therefore, in our simulations, the probability that a beneficial mutation escapes drift is likely higher than in the experiments (although similar to the expected from Wahl et al. 2002 and dependent on *s*; see Supplementary Fig. S9). The immediate consequence of a decrease in genetic drift is that we underestimate the beneficial mutation rate. There may also be changes to the distribution of contending mutations which will in turn affect how well each model fits to the data. However, the alternative of using a constant population would have more drastic consequences on the distribution. In order to overcome these problems, new efficient simulation tools need to be developed and validated, preferably against measures of drift in experimental systems.

Given the pervasive nature of diminishing returns epistasis for beneficial mutations, it is reasonable to assume that a general model exists that can explain and predict adaptation across different systems. Since epistasis is the result of specific constraints to biological systems, this implies that there are common rules in how biological systems work (in line with the results obtained in Chiu et al. 2012). While the power law model we tested explains the experimental results well, the best set of parameters estimated for each dataset differs in all scenarios. We suggest two different interpretations for these relatively arbitrary sets of parameters. The first hypotheses is that indeed, each species in each condition will behave as the model predicts, but without any relation to other species or conditions. This would strongly decrease our ability to extrapolate knowledge obtained in experiments to predict new data. Our second hypothesis is that, given the complex multidimensional space defined by the model parameters, we might have only found a local maximum of our score function. This would mean that there might exist a region of parameter space that explains all datasets well but that we have not been able to find it despite our best efforts. As is common in biology, these two hypotheses are not mutually exclusive. It is possible that in each dataset, given the specificities of the species, genotypes, and conditions selected, the best possible set of parameters that reproduce the observed behavior is unique to this set. However, some potentially general set of parameters or parameters combinations might be able to also reproduce the data within reasonable bounds.

In order to test the predictive power of each model, we tested whether they could predict the dynamics on unobserved data. We started with extra data from the LTEE, where the model predicted an adaptation rate and rate of fixation within the observed values. While fitness was generally well predicted, there was a significant overestimation of the fixation rate (Fig. 2c). This could be due to the presence of other types of selection such as frequency-dependent selection, which our model does not capture and which have been observed in the later stages of the LTEE (Good et al. 2017). We also tested if the models could predict microscopic epistasis from the LTEE. We found that the power law model captures the overall trend of diminishing returns for each individual mutation. For the budding yeast dataset we also tested how good each model was at predicting the fitness effect of individual mutations. We found that the power law could only explain 62% of the observed variation in change in selection coefficient with background. Since only a few mutations (all gene knockouts) were tested, it could be that they are not a representative subset of the adaptation experiment.

Previous work has attempted to fit diminishing returns epistasis patterns in similarly simple models. In particular, Wiser et al. (2013) found that a power law model fits the LTEE better than a model with a maximum. Here, we expand on their analysis and show that the same model that explains the experimental evolution can also capture the patterns of microepistasis observed when single mutations are taken and tested on different backgrounds (Fig. 3). Moreover, we extend their analysis to other datasets. Recently, Blanquart and Bataillon (2016) used FGM to show that it can predict microepistasis in some cases, but not in others. In a similar manner, Good and Desai (2015) have analyzed patterns of micro and macroepistasis in the LTEE and conclude that a two-epoch model is needed to explain all of the trajectory of the LTEE. Here, we show that by reformulating the power law model into a commutative model, we do not need two epochs to reasonably explain the later time points of the experiment with only information on the first 10,000 generations (Fig. 2c). There is, however, some biases in the residuals of our prediction and a recent publication has shown that there is a different type of dynamics in the latter part of the experiment (Good et al. 2017). More work is needed to understand how much we can predict on LTEEs and whether or not we need to invoke different rules as hundreds of mutations accumulate.

In this work we focused on a simplified formulation of fitness landscapes. The power law model is effectively one dimensional on the phenotypic level, which we expect to represent multiple underlying biological phenotypes contributing to fitness. Multidimensional phenotypic landscapes, namely, FGM (Fisher 1930) received a lot of attention in the literature and is regarded as a standard evolutionary model (Tenaillon 2014). Although FGM can explain several patterns in adaptation, here we show that if we reduce FGM to a comparable simplicity to the power law, the latter is better at describing experimental data than the former. Of course, we cannot exclude that other formulations of the model would outperform the power law. Importantly, FGM predicts that beneficial mutations eventually overshoot the optimum phenotype level, becoming deleterious. In the microscopic epistasis datasets analyzed here there is no evidence for this sign epistasis.

We note that across many systems there are several examples of sign epistasis, i.e., of mutations that are both beneficial and deleterious depending on the genetic background. Except for FGM, none of our models allows for a mutation to have a deleterious effect in any background. This makes them unsuitable to describe populations where deleterious mutation are expected to fix, such as mutation accumulation experiments (e.g., Kibota and Lynch 1996). It is not, however, clear whether we need the full spectrum of mutations to explain the evolution of populations under strong positive selection.

To put into perspective how good our predictions are, we tested the performance of each model using only a portion of the experimental data. We show that the power law is able to predict fitness, even with small datasets. Moreover, we can use data from adaptation experiments (macroepistasis) to predict the fitness effects of single mutations across backgrounds (microepistasis). This might be relevant, for example, when building transgenics using genes that resulted from evolution experiments.

In this work we found that a phenotype-fitness landscape shaped like a power law can satisfactorily explain and predict patterns of adaptation in different microorganisms. This observation implies that, at least in the laboratory, microorganisms are very far away from their optimum and may not reach it at all since with time it is expected that the fitness landscape might change radically (Blount et al. 2008) or new interactions might emerge (Good et al. 2017).

## Electronic supplementary material

Supplementary Figure 1

Supplementary Figure 2

Supplementary Figure 3

Supplementary Figure 4

Supplementary Figure 5

Supplementary Figure 6

Supplementary Figure 7

Supplementary Figure 8

Supplementary Figure 9

Supplemental File 1

Supplemental File 2 - Fitness Fission Yeast

Supplemental File 3

Supplemental Tables
