# Supplementary figures and images for: Power law fitness landscapes and their ability to predict fitness
Source: Heredity (Edinb). 2018 Sep 6;121(5):482–98. doi: 10.1038/s41437-018-0143-5 (PMC6180038; doi:10.1038/s41437-018-0143-5)

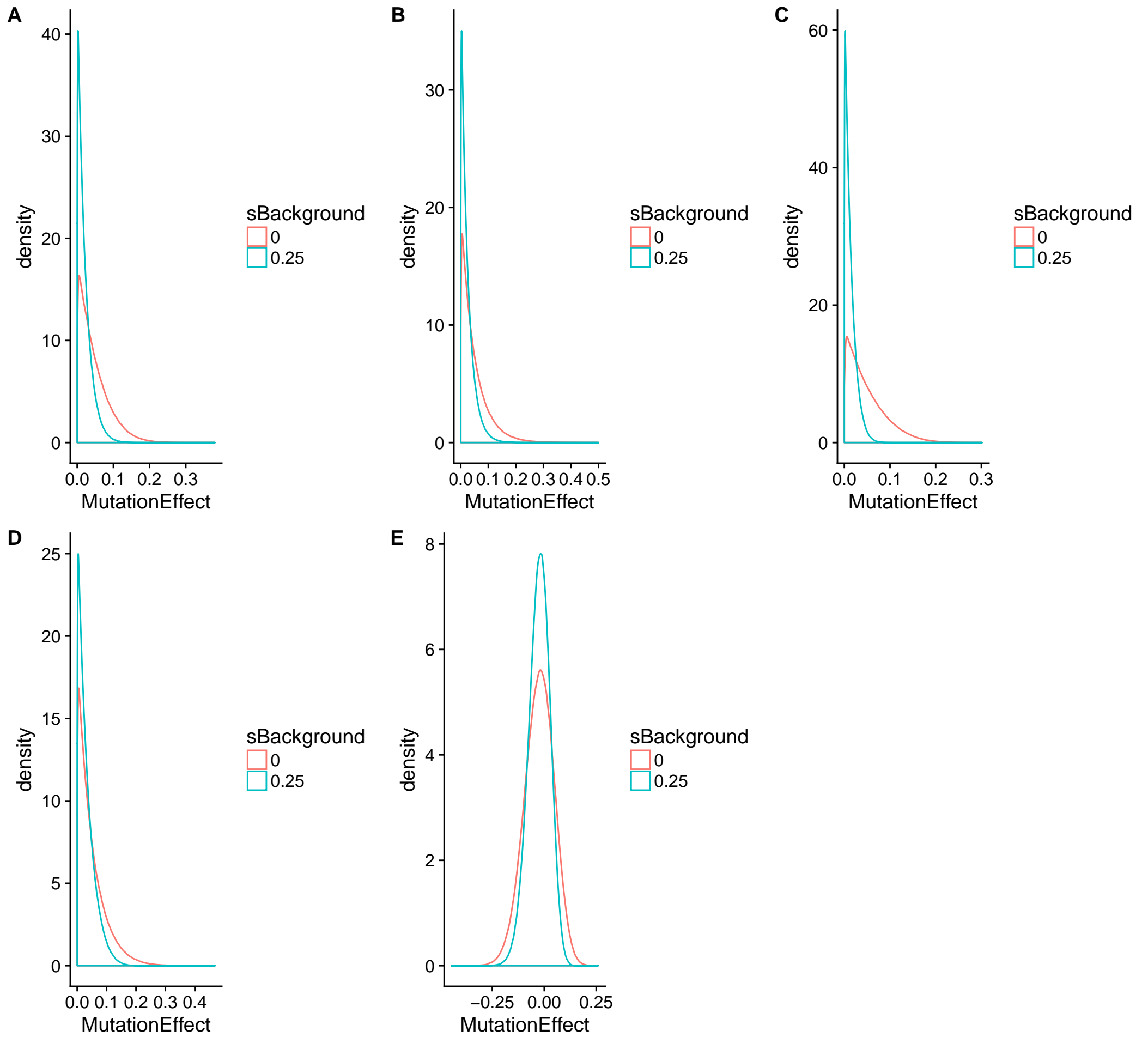

Supplement: Supplementary file 1 — Supplementary Figure 1 [file 41437_2018_143_MOESM1_ESM.pdf]

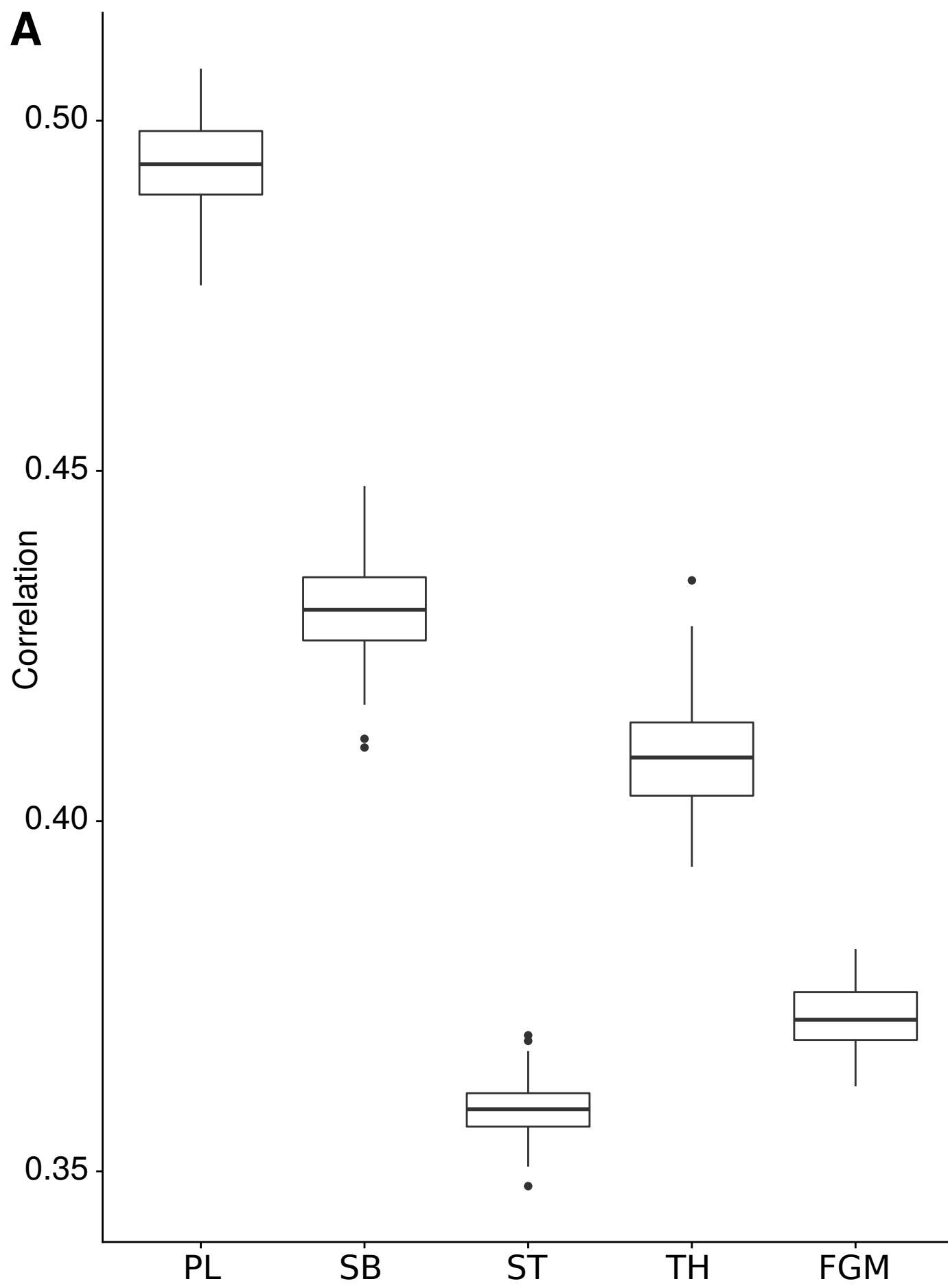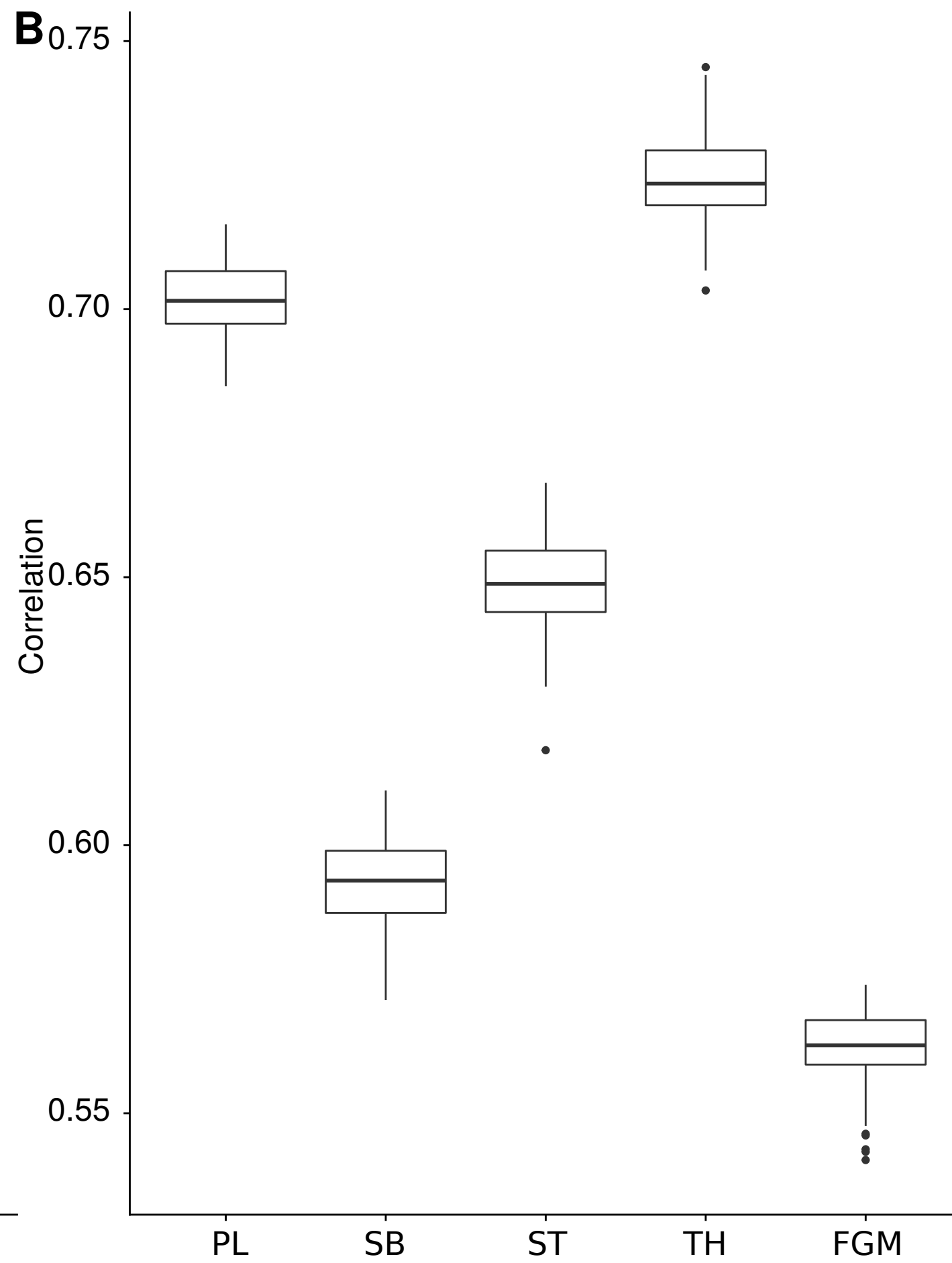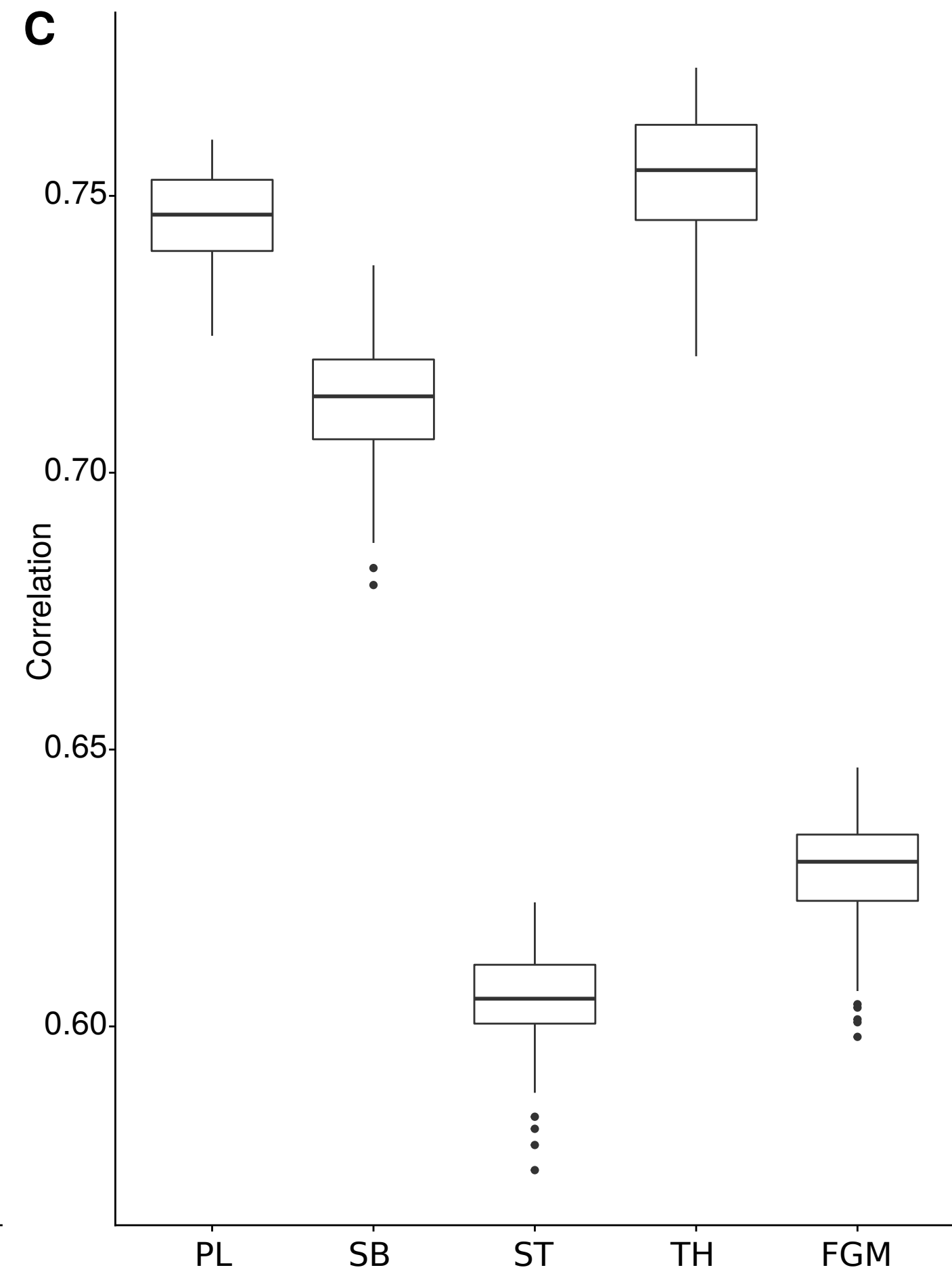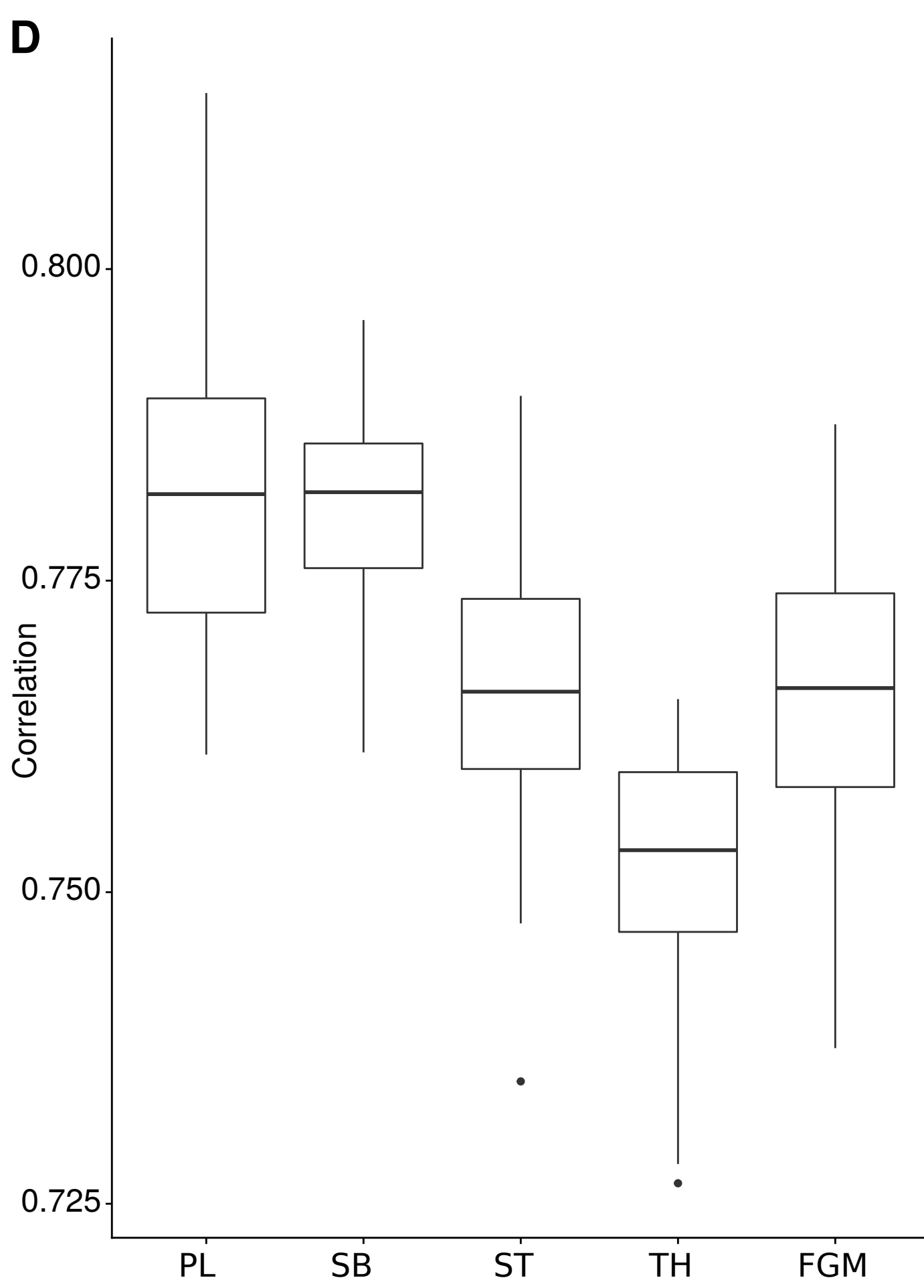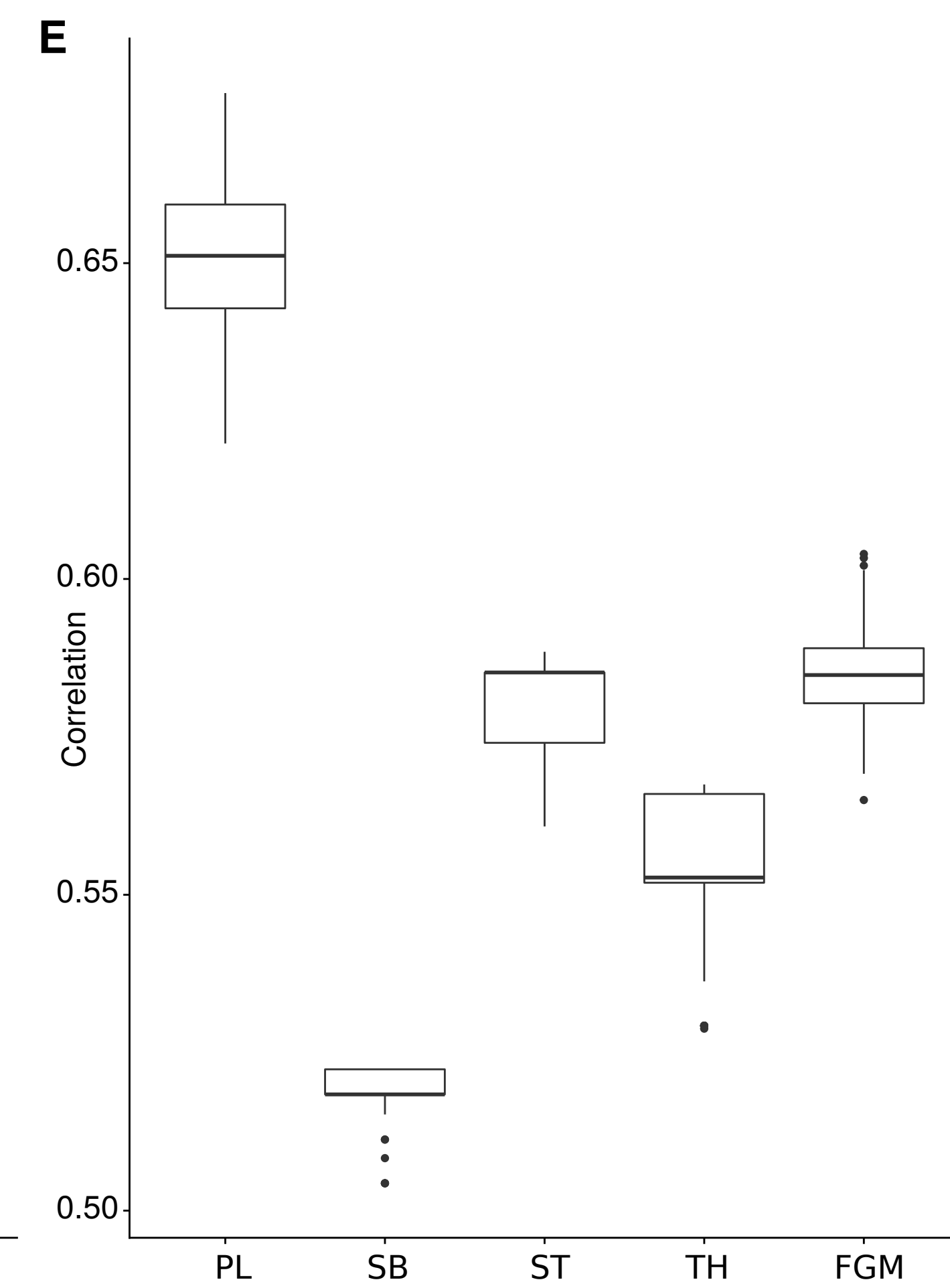

Supplement: Supplementary file 2 — Supplementary Figure 2 [file 41437_2018_143_MOESM2_ESM.pdf]

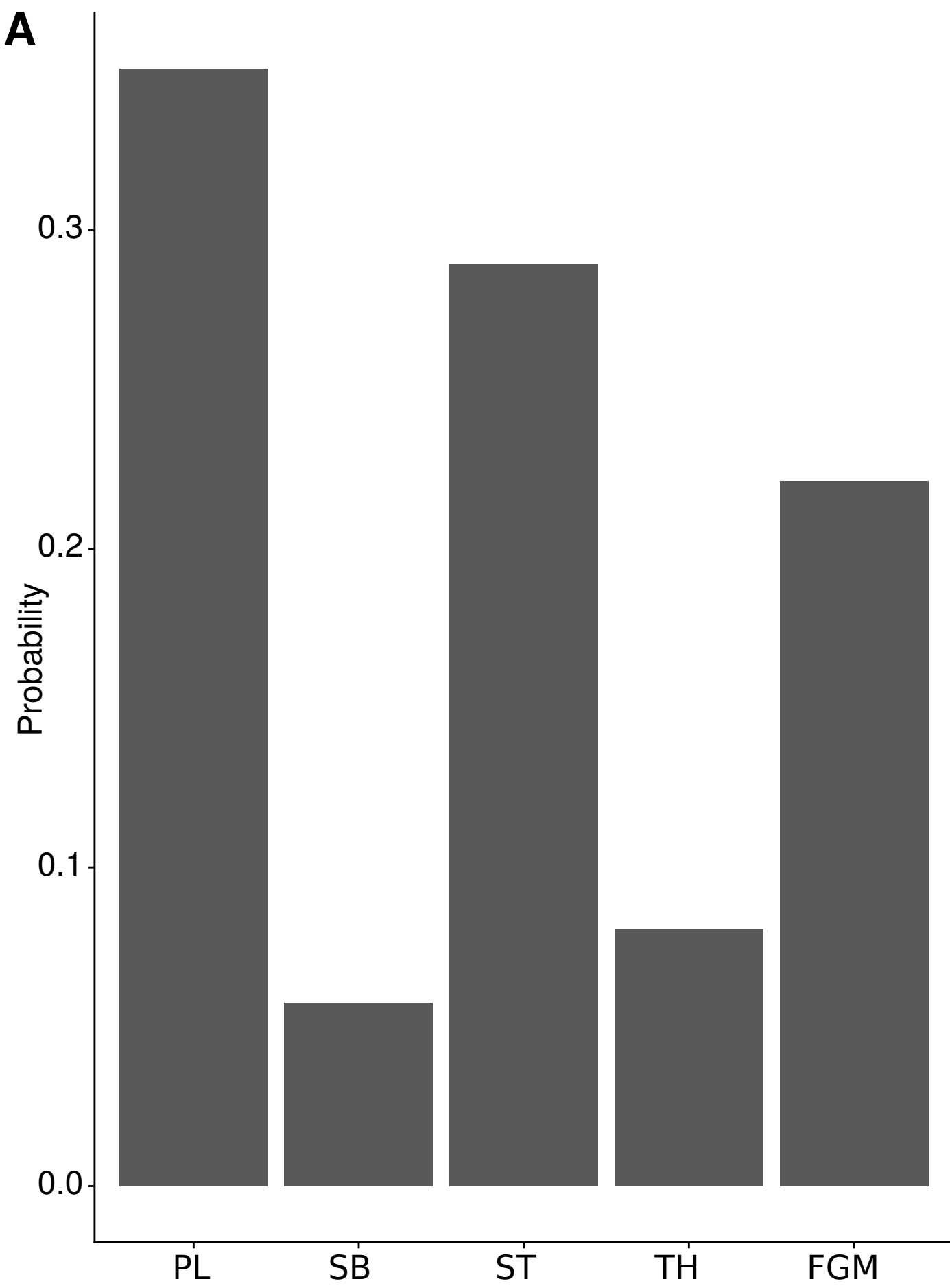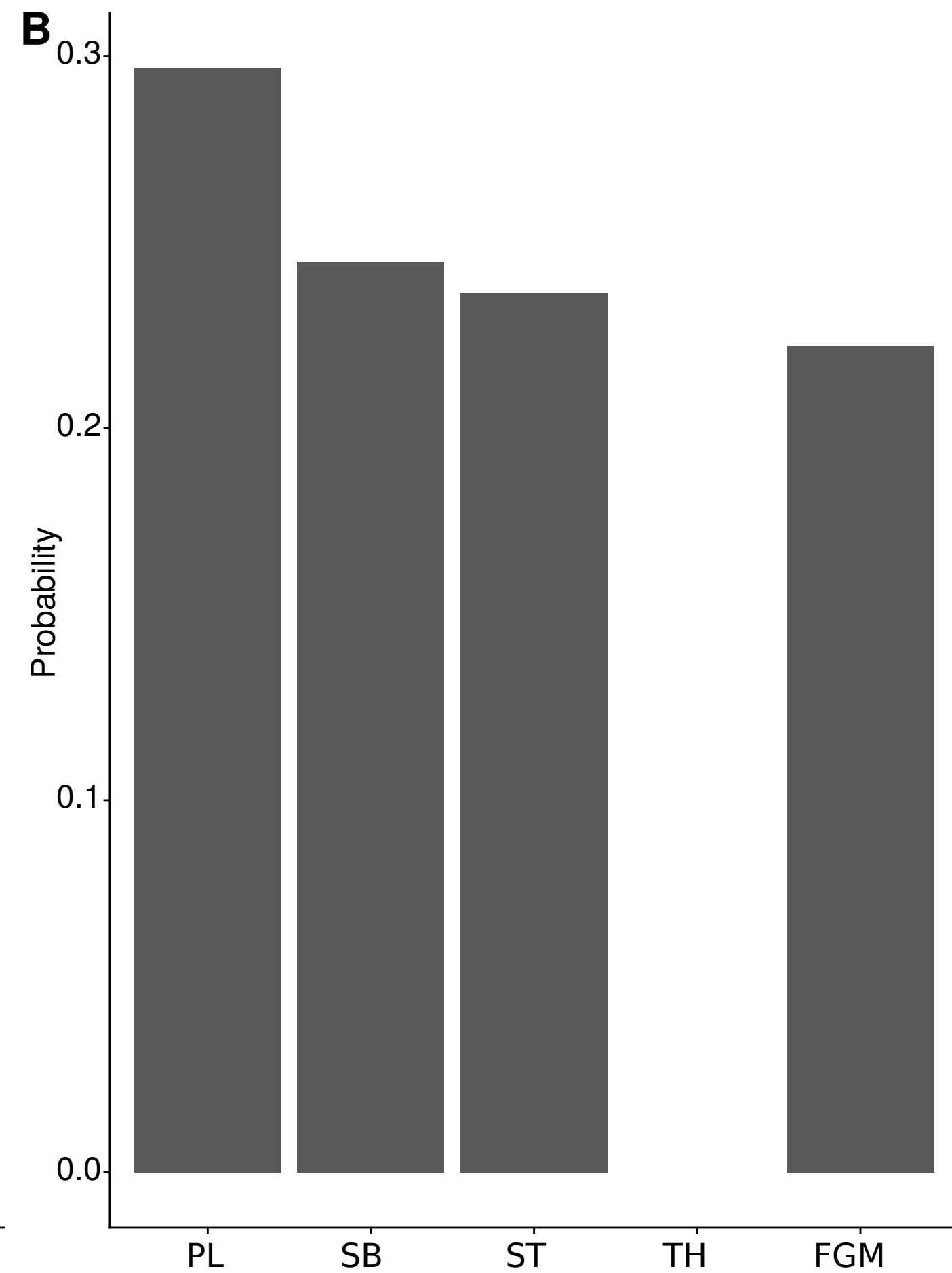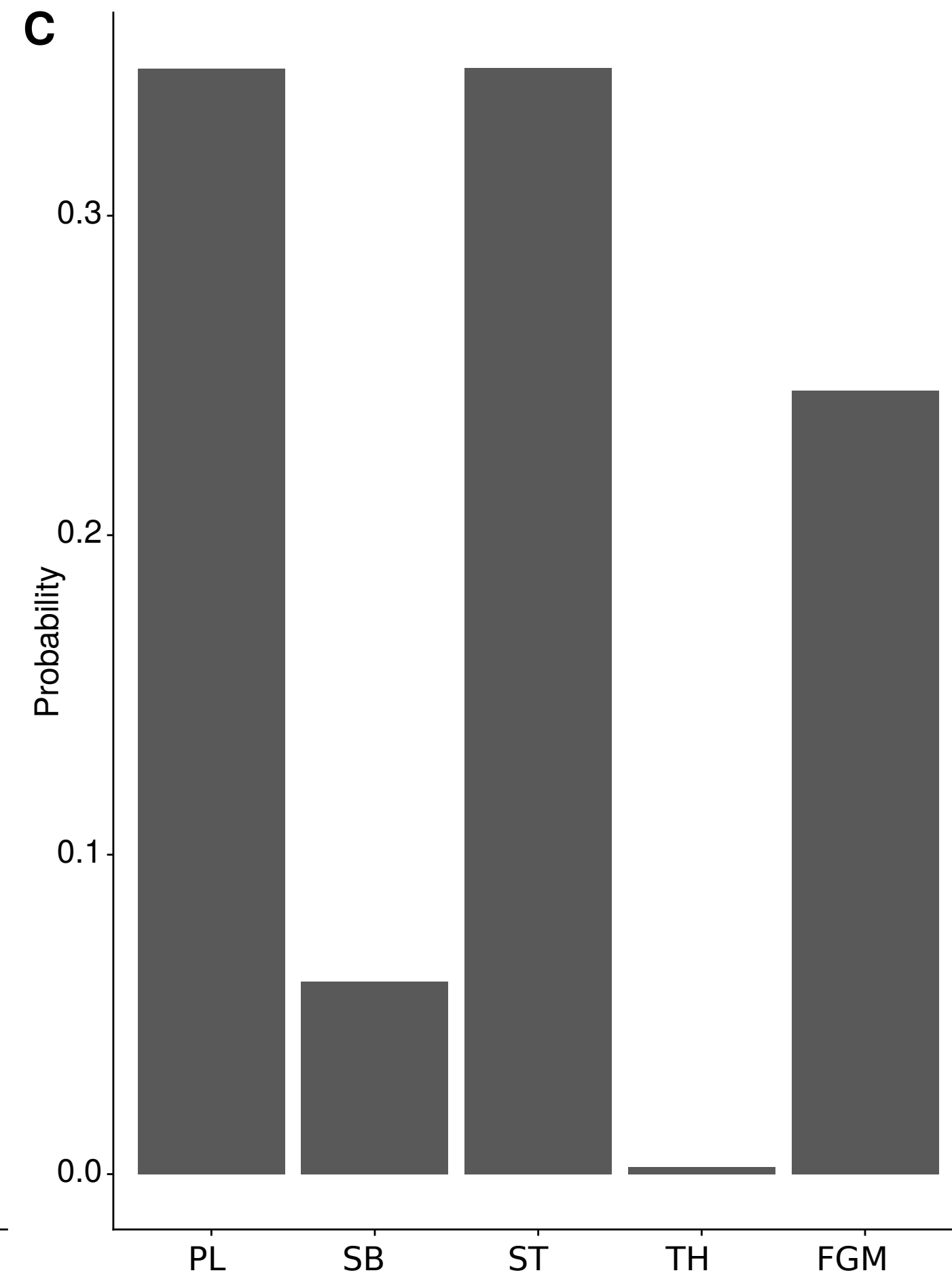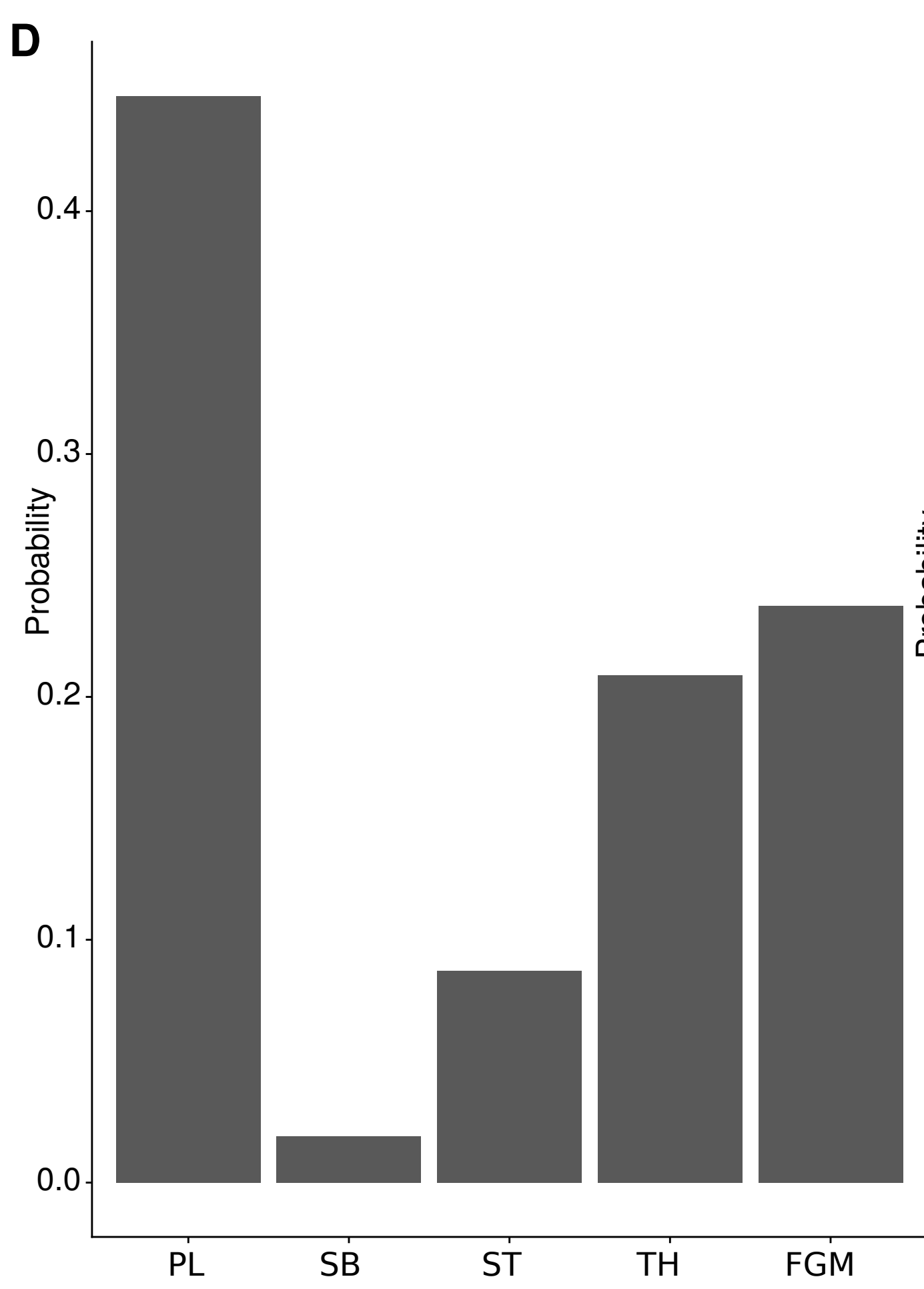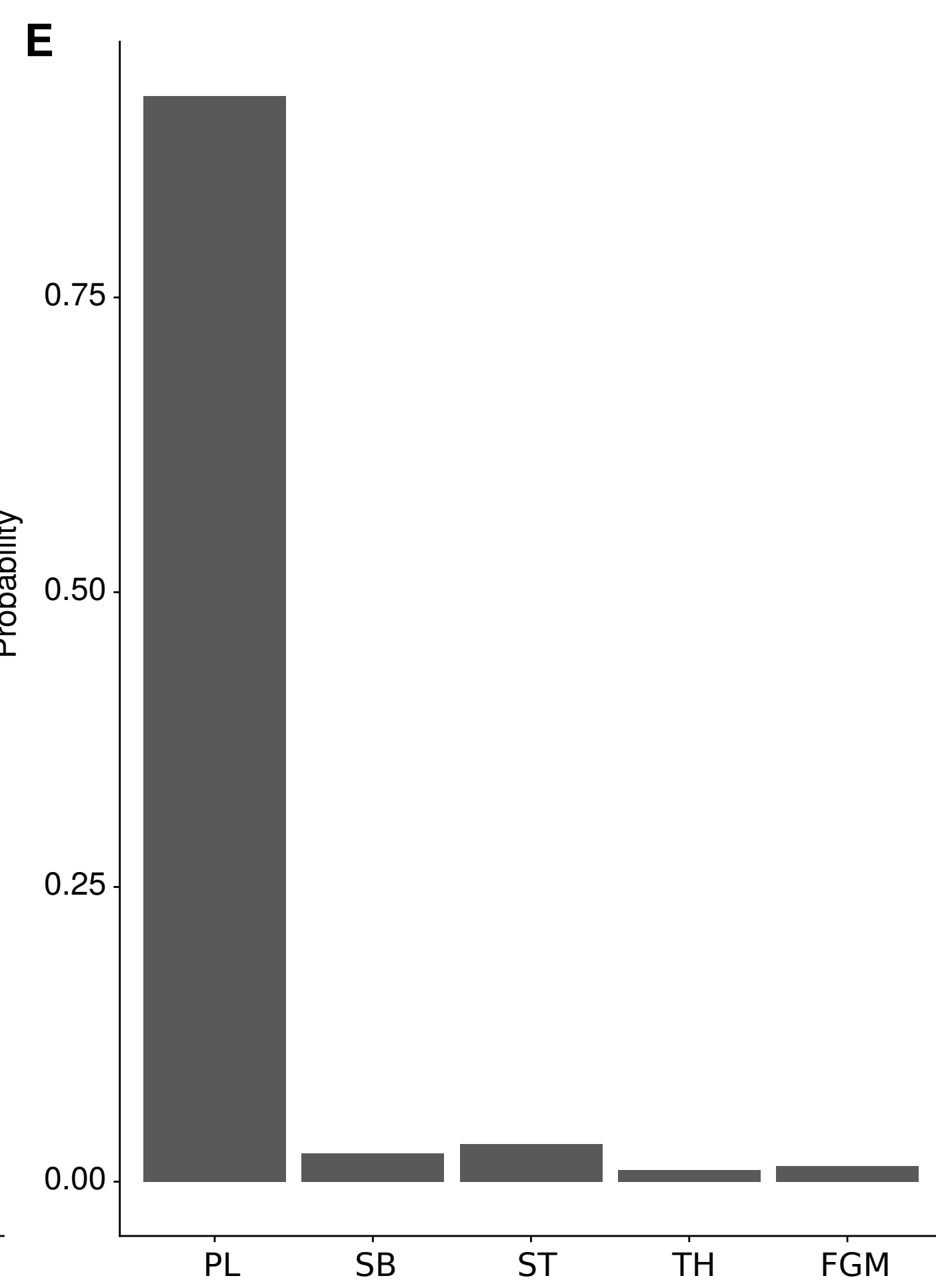

Supplement: Supplementary file 3 — Supplementary Figure 3 [file 41437_2018_143_MOESM3_ESM.pdf]

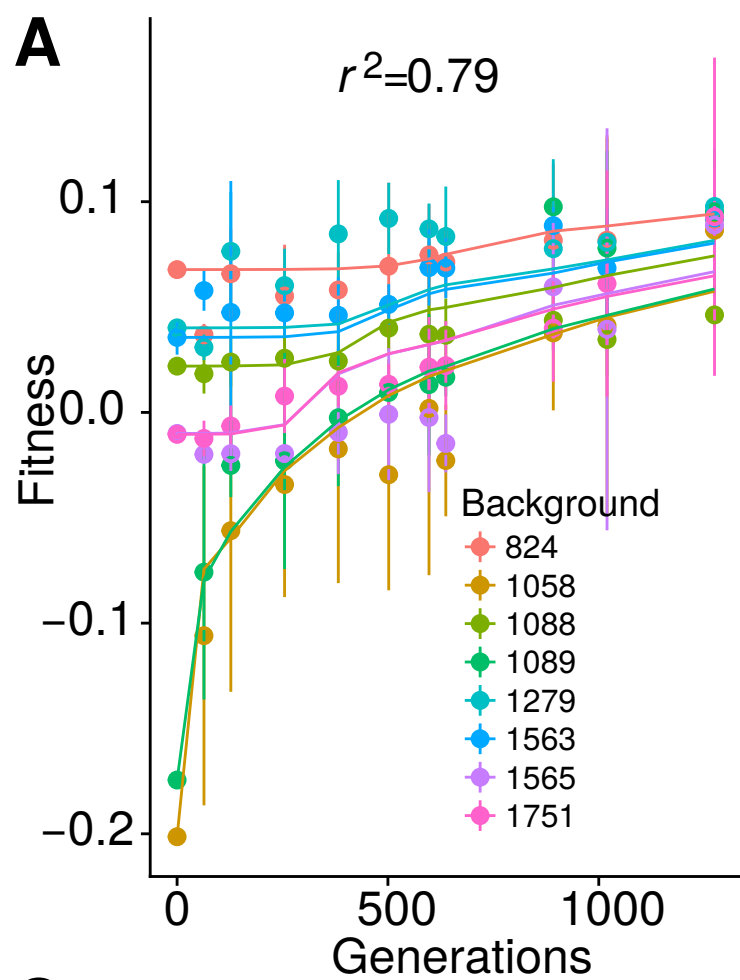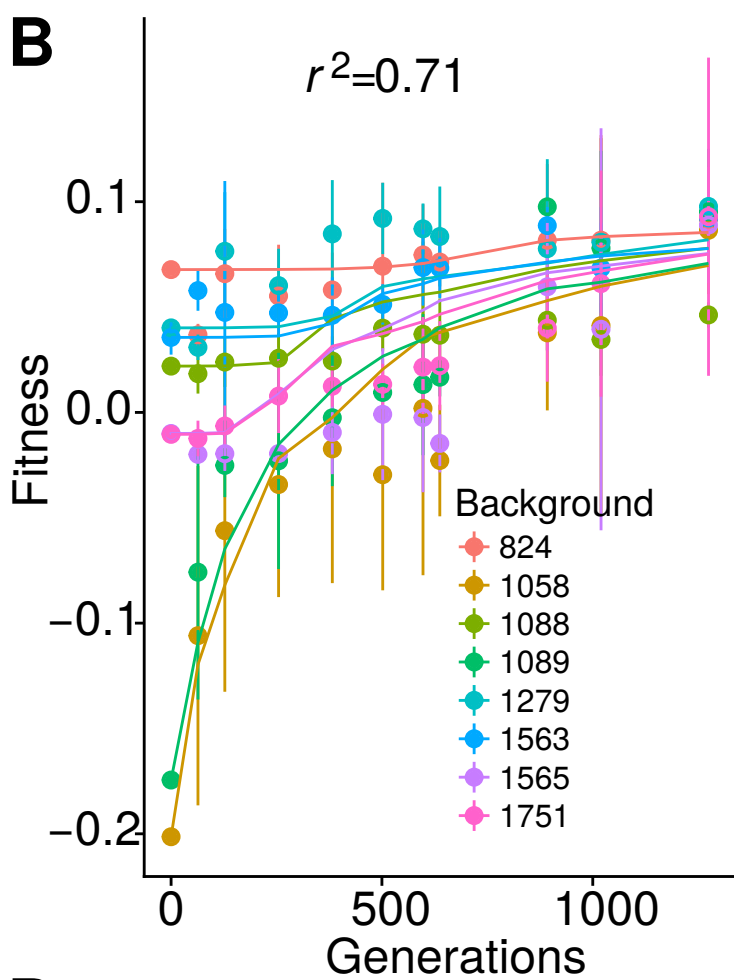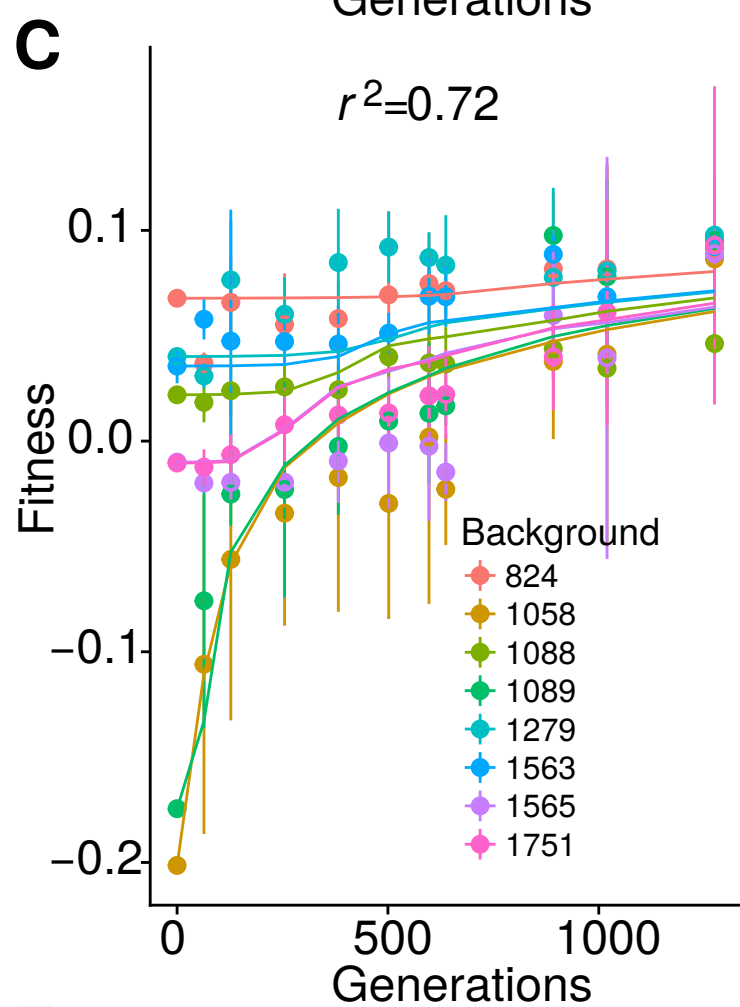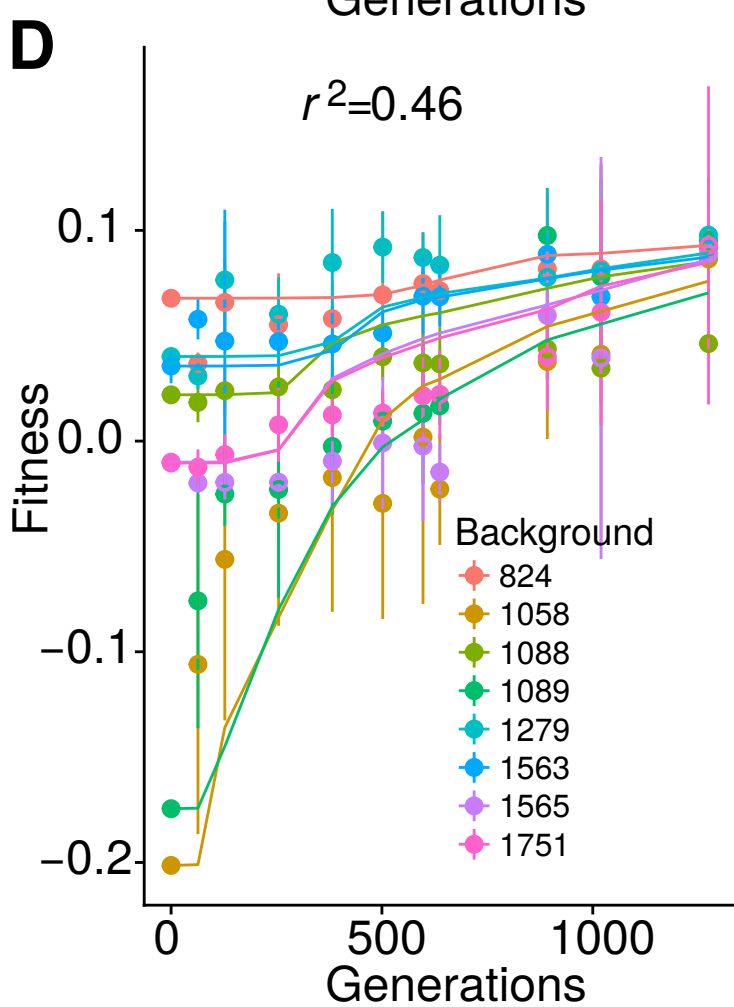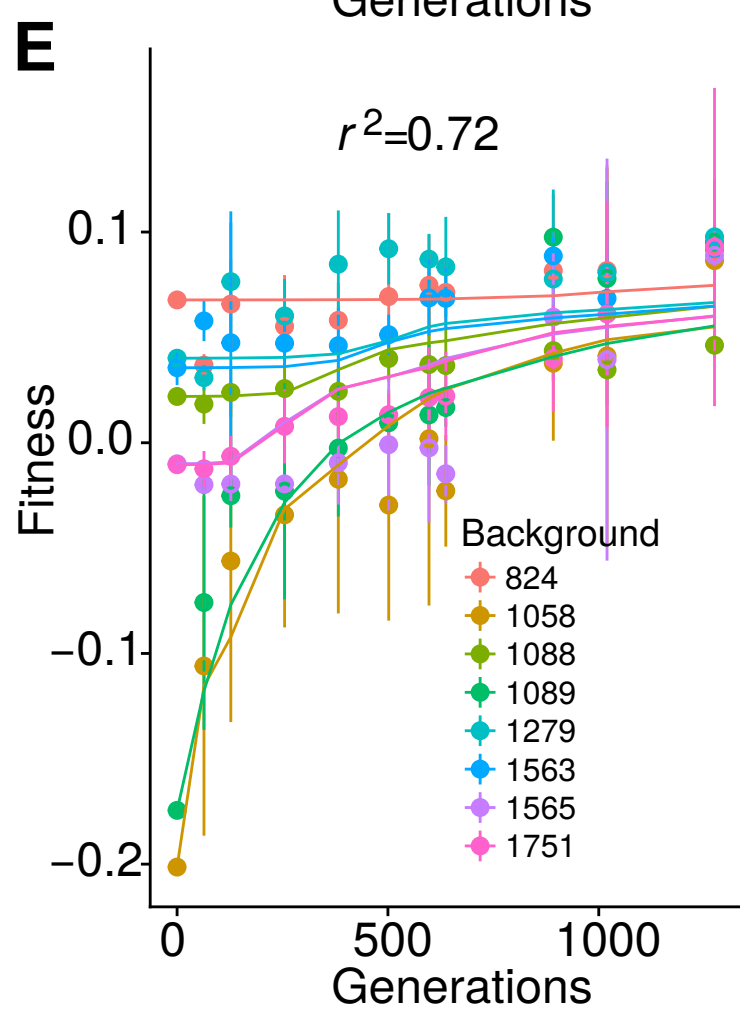

Supplement: Supplementary file 4 — Supplementary Figure 4 [file 41437_2018_143_MOESM4_ESM.pdf]

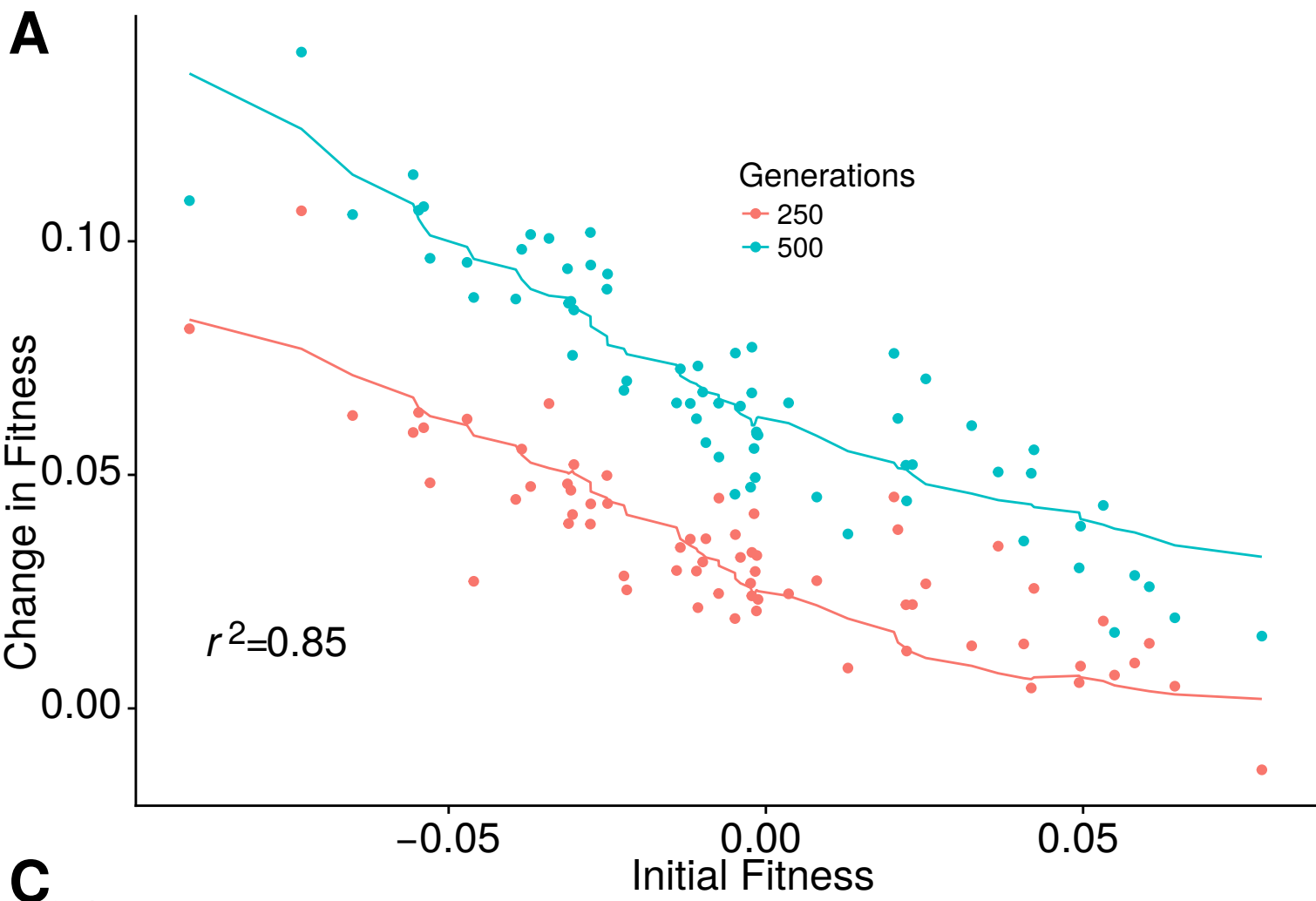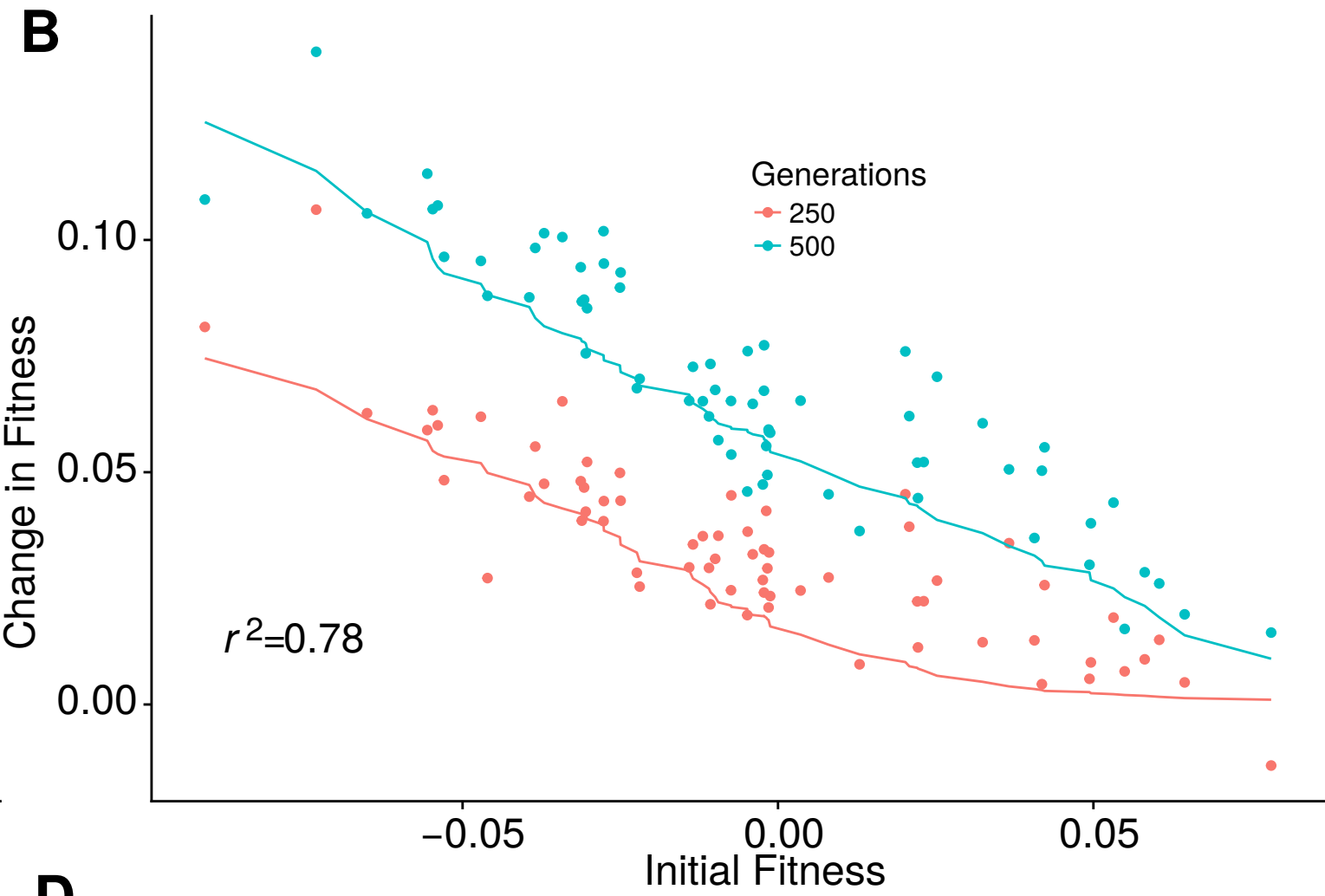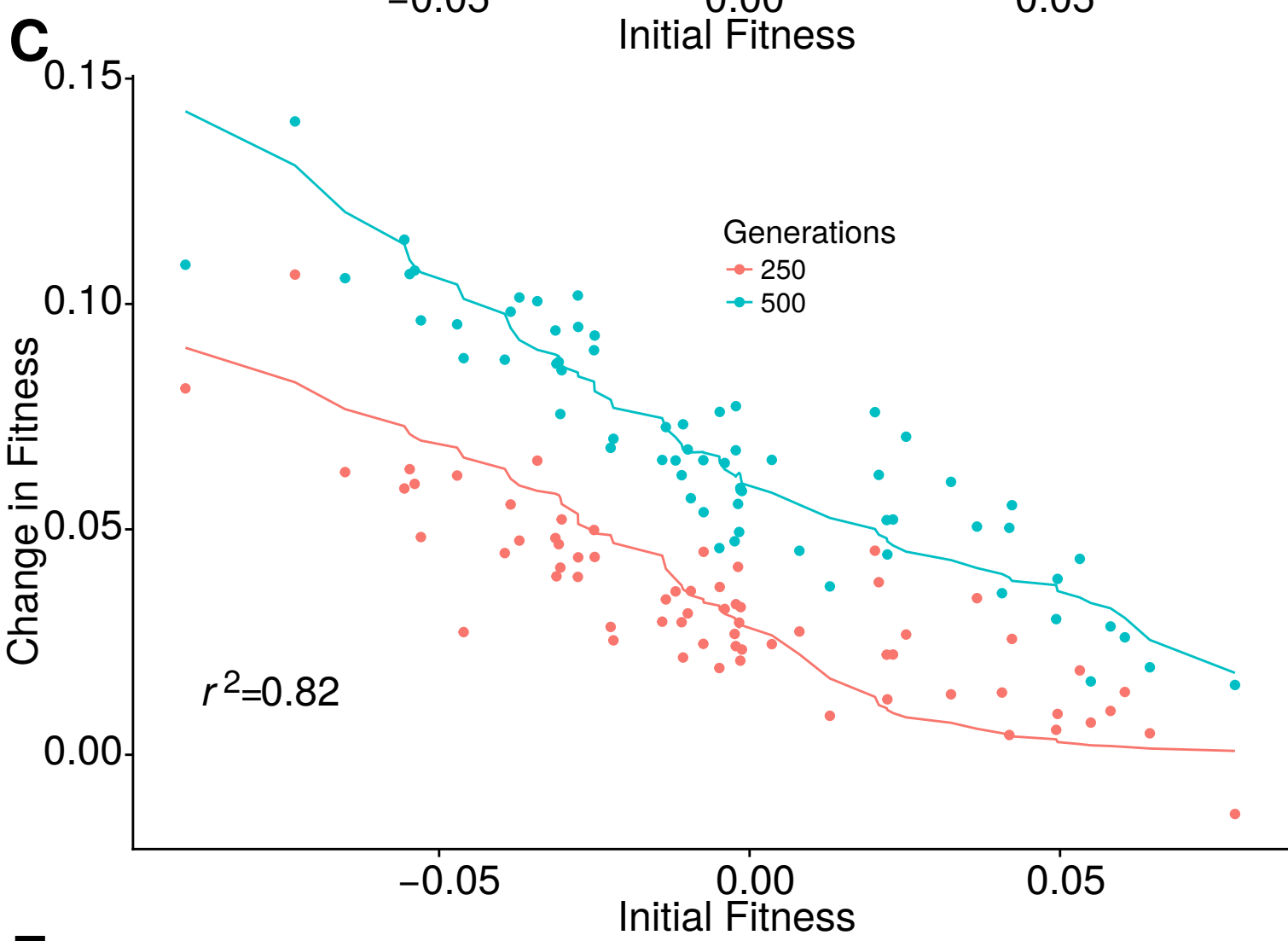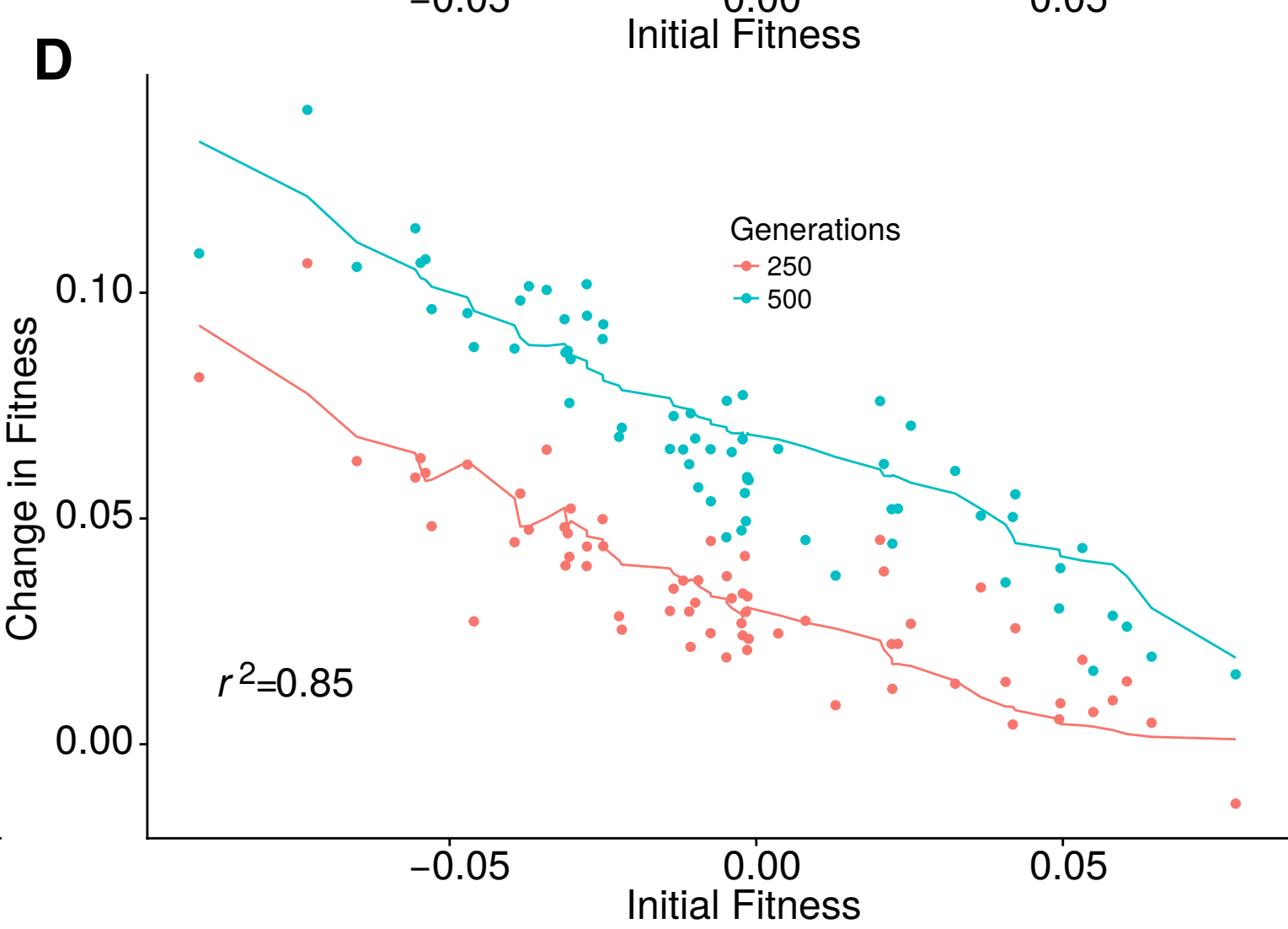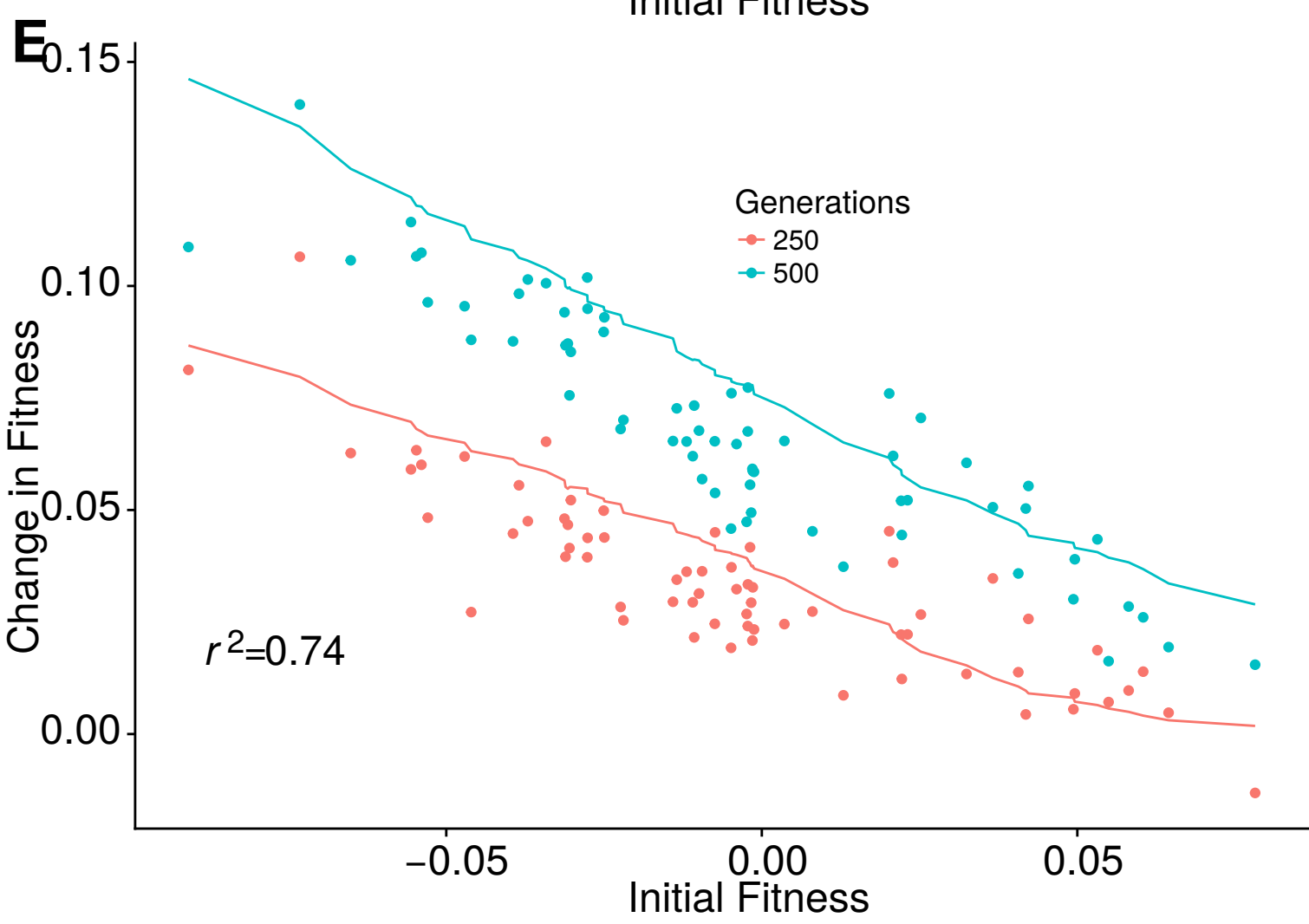

Supplement: Supplementary file 5 — Supplementary Figure 5 [file 41437_2018_143_MOESM5_ESM.pdf]

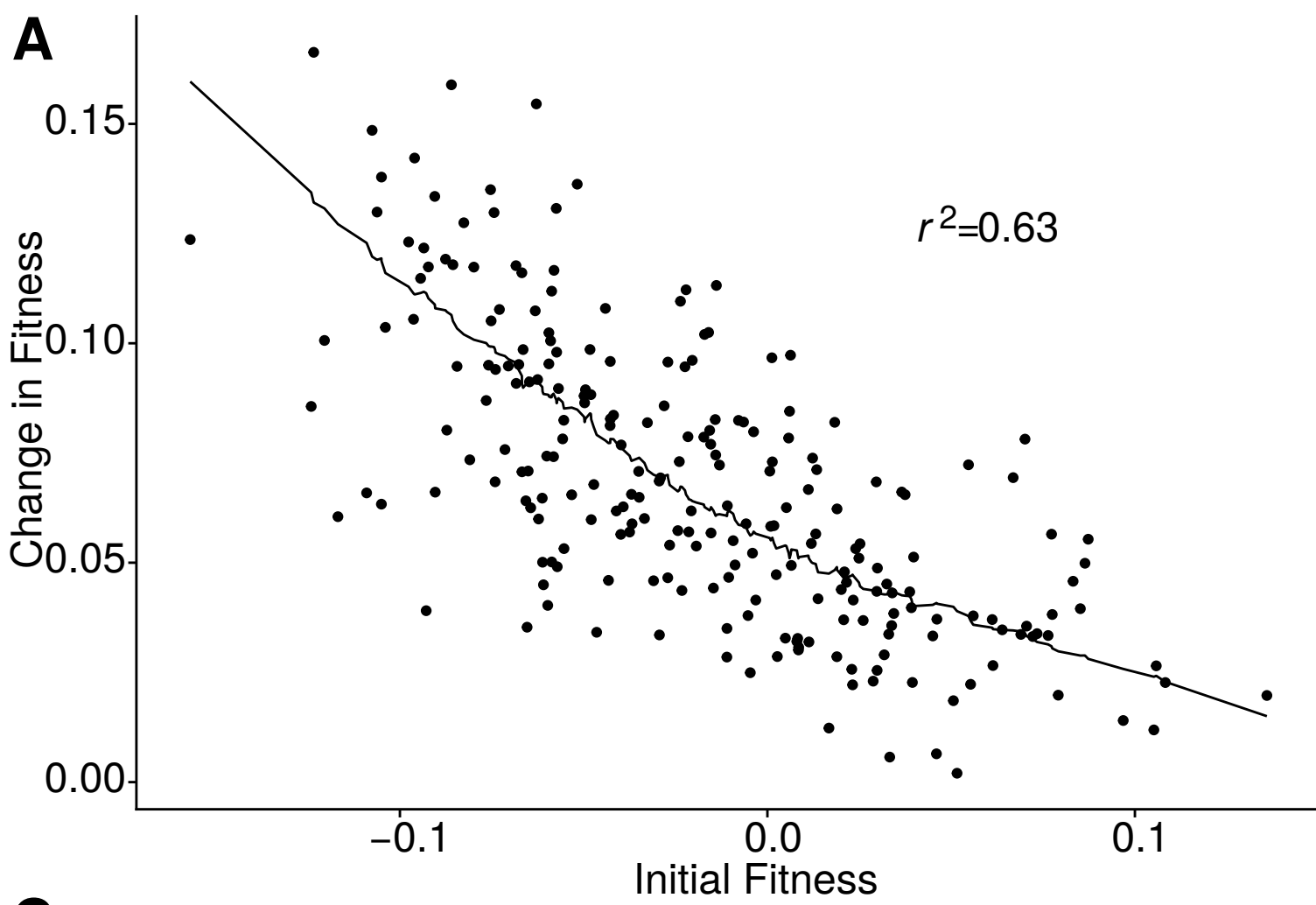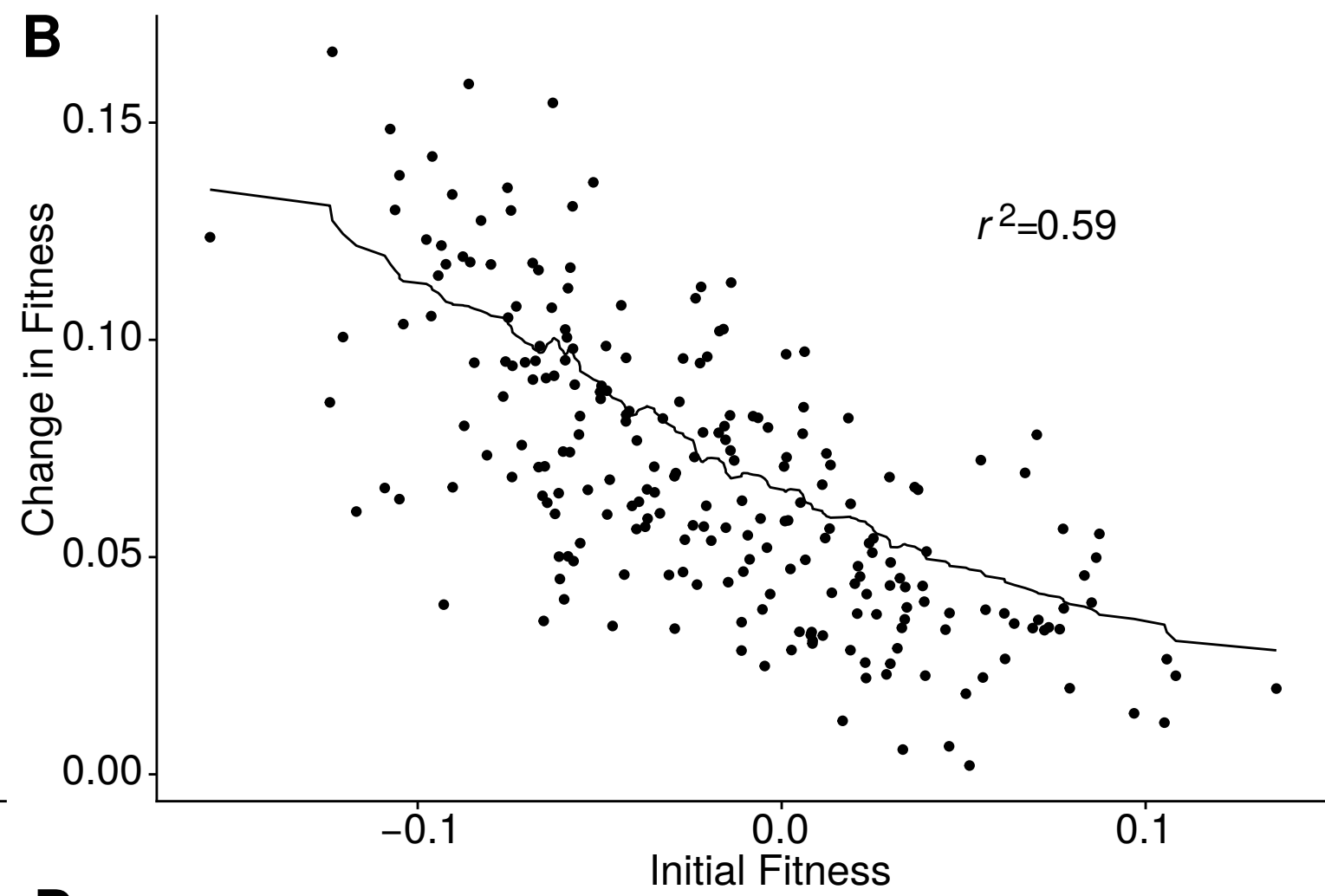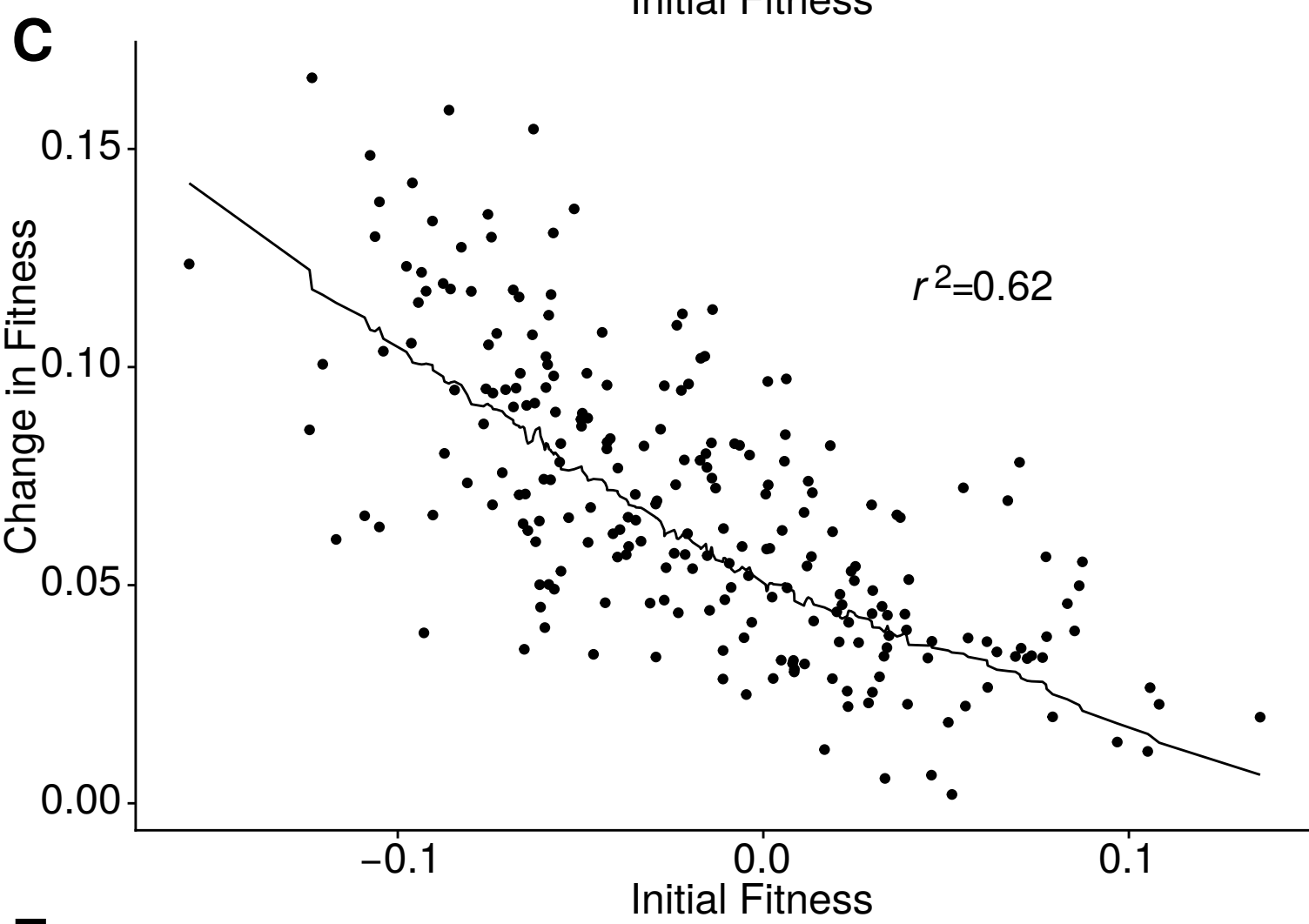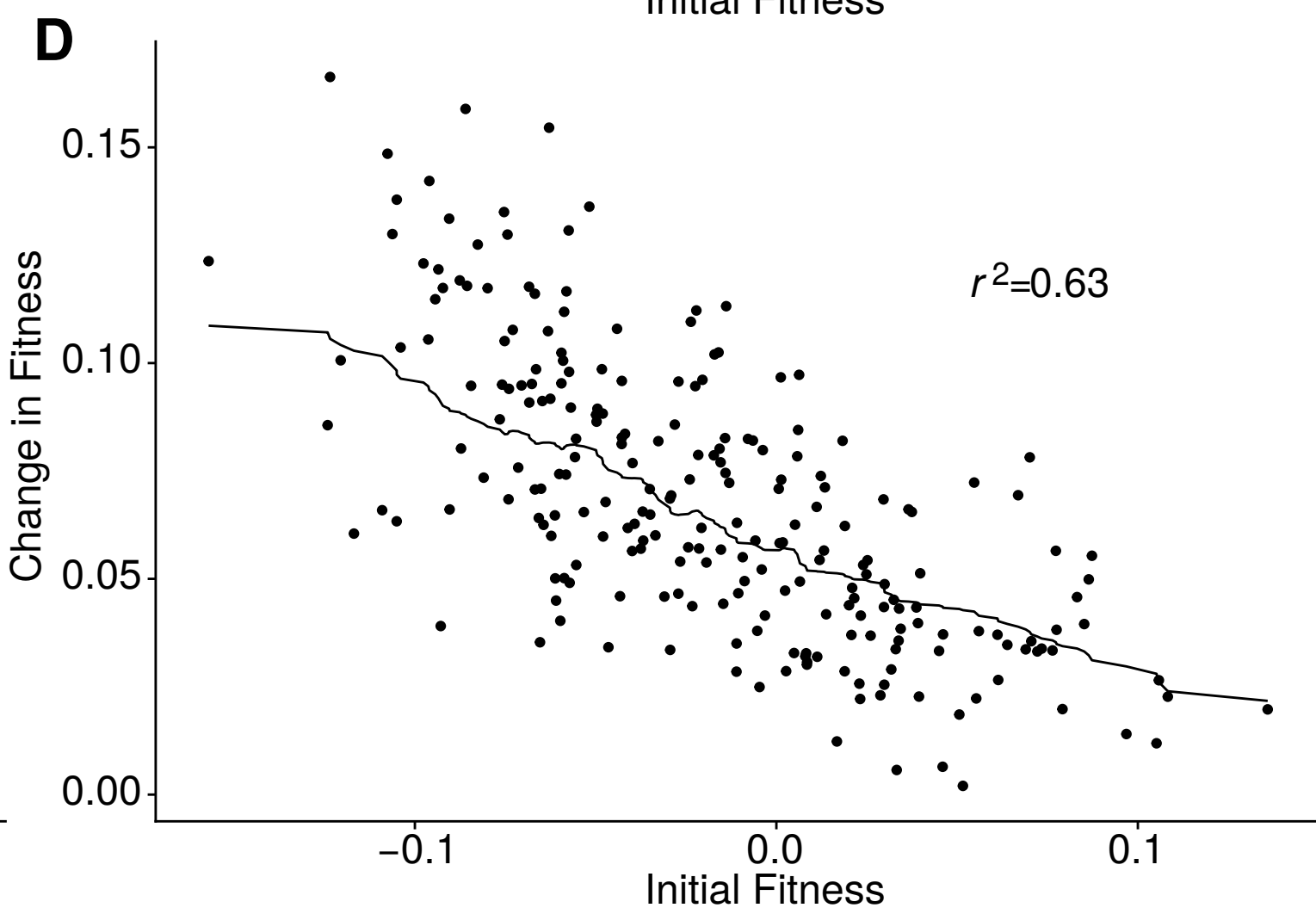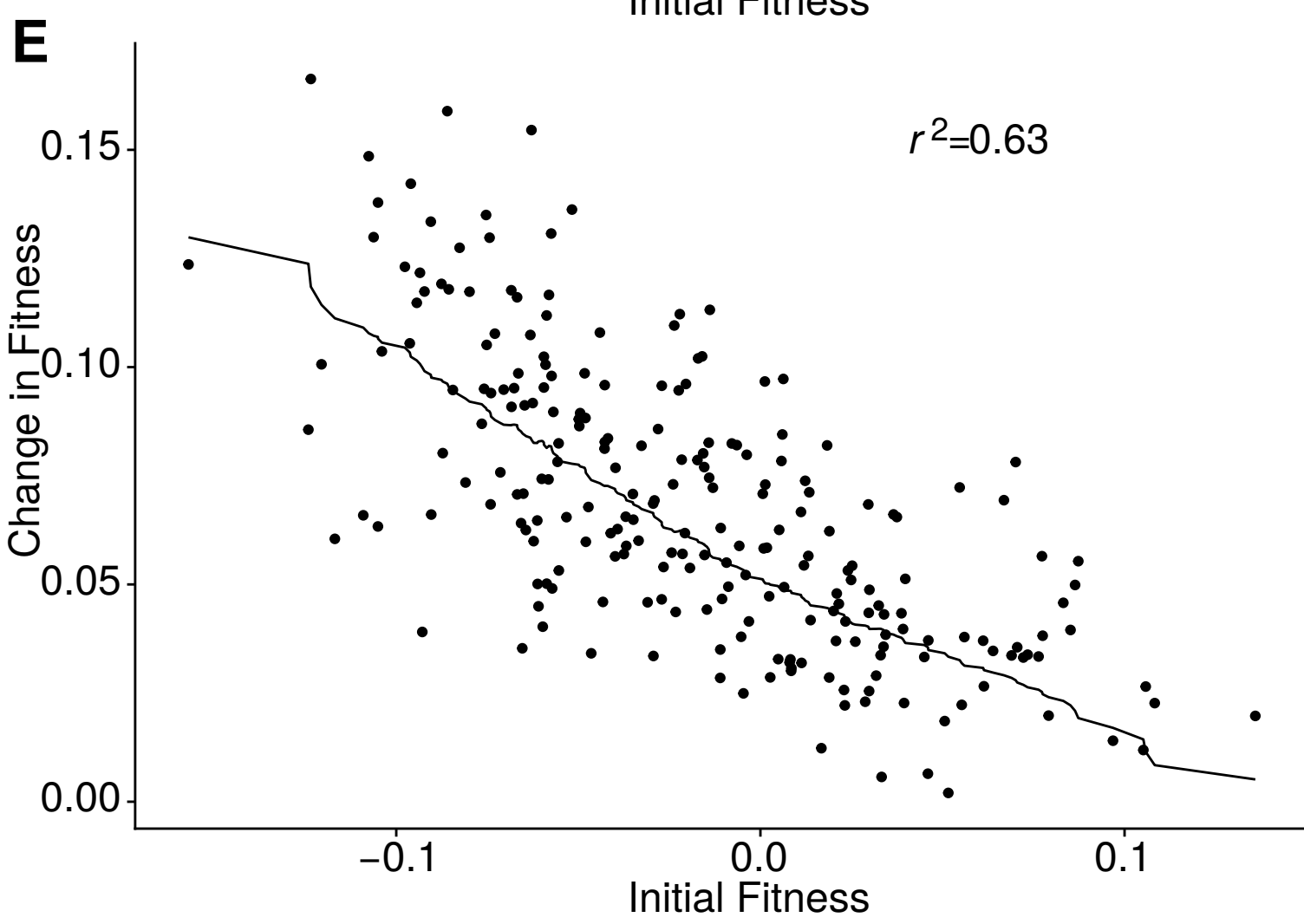

Supplement: Supplementary file 6 — Supplementary Figure 6 [file 41437_2018_143_MOESM6_ESM.pdf]

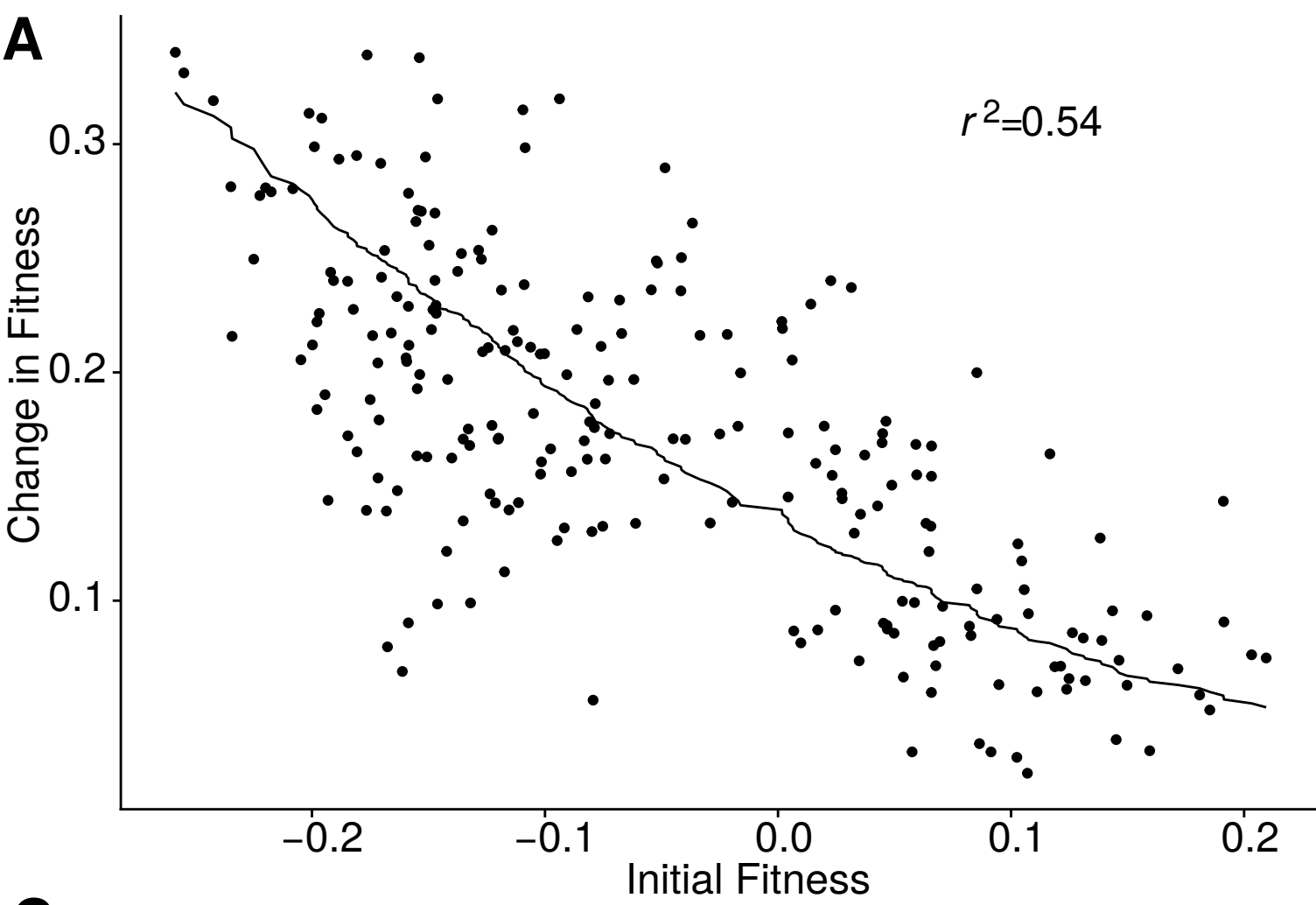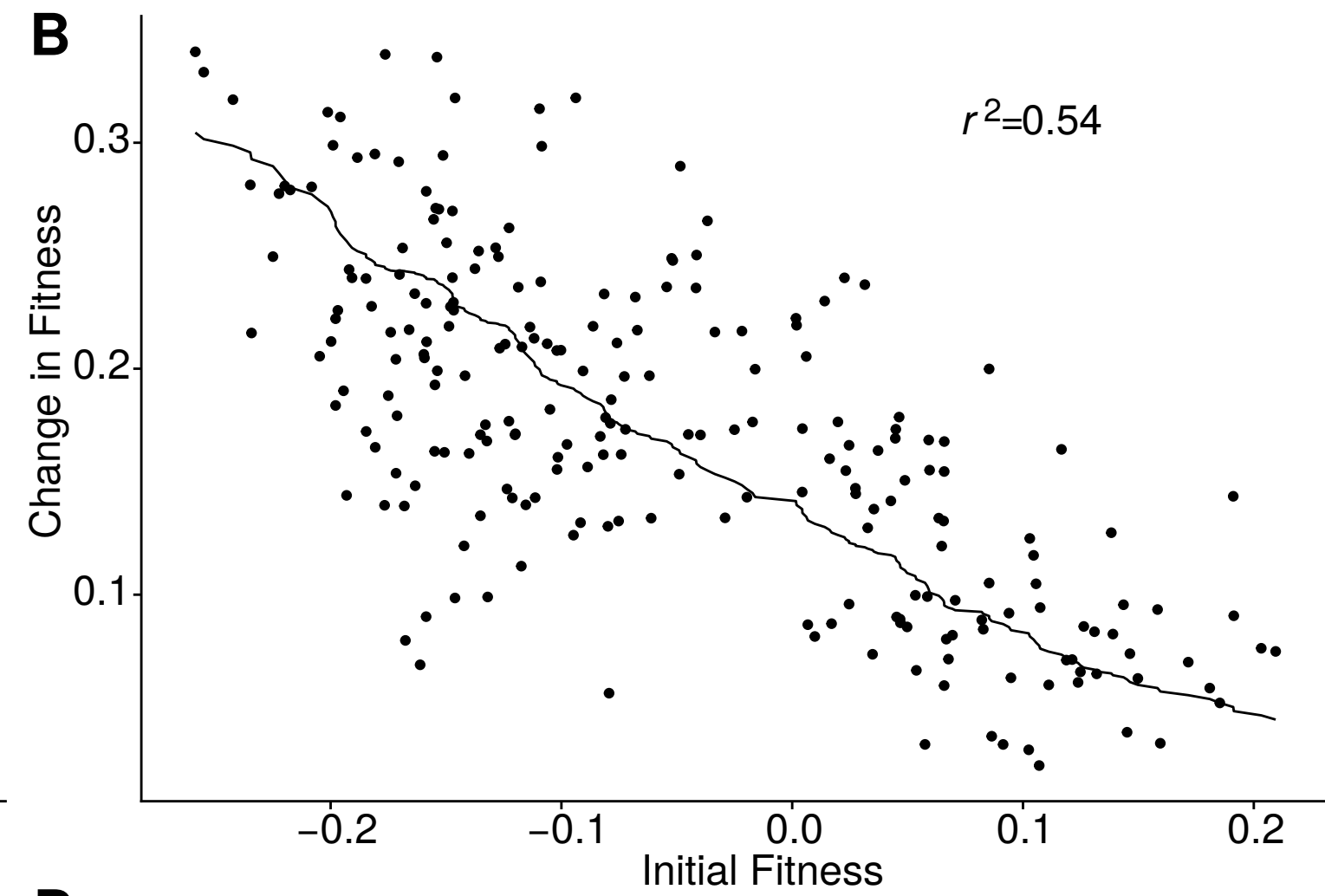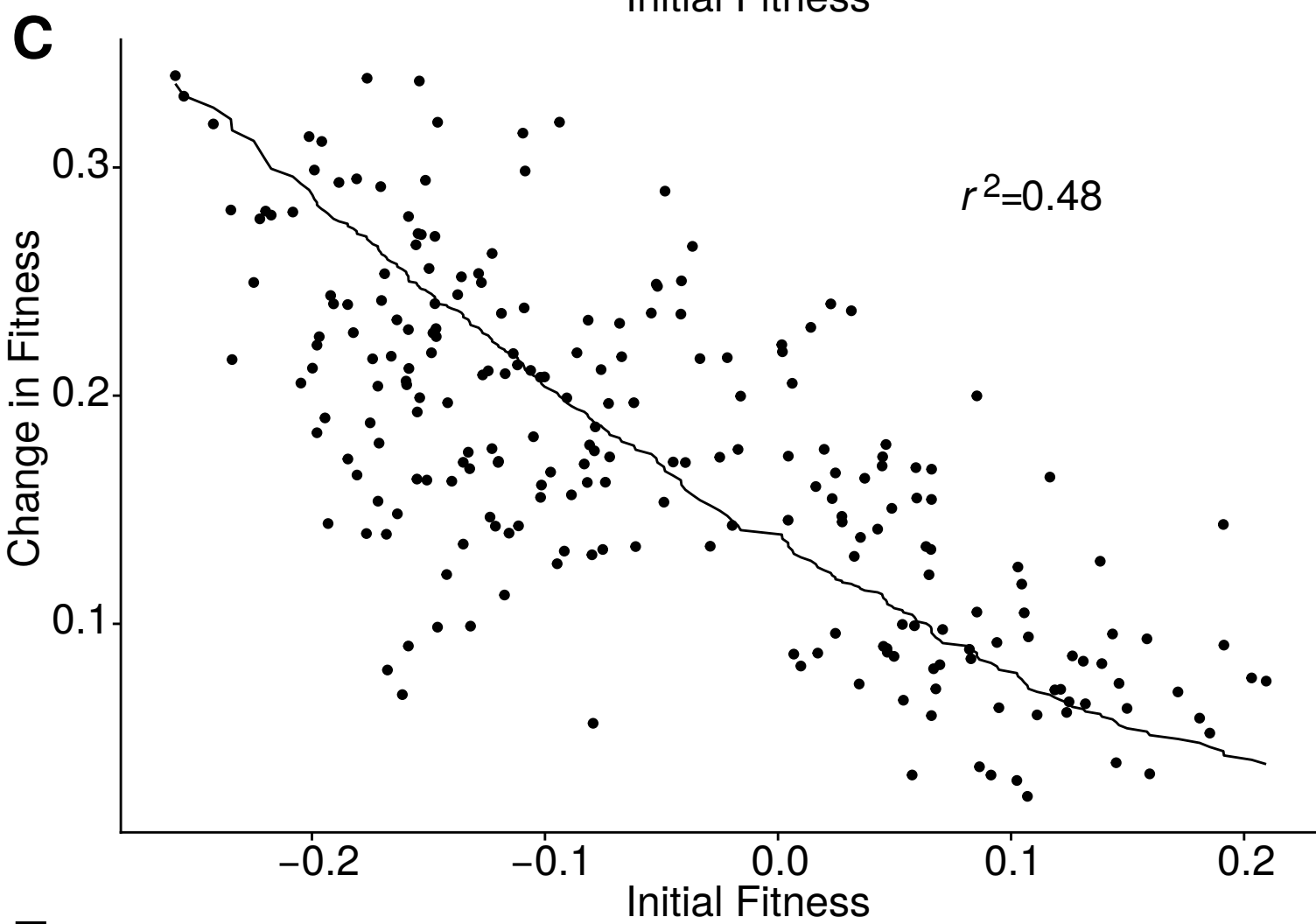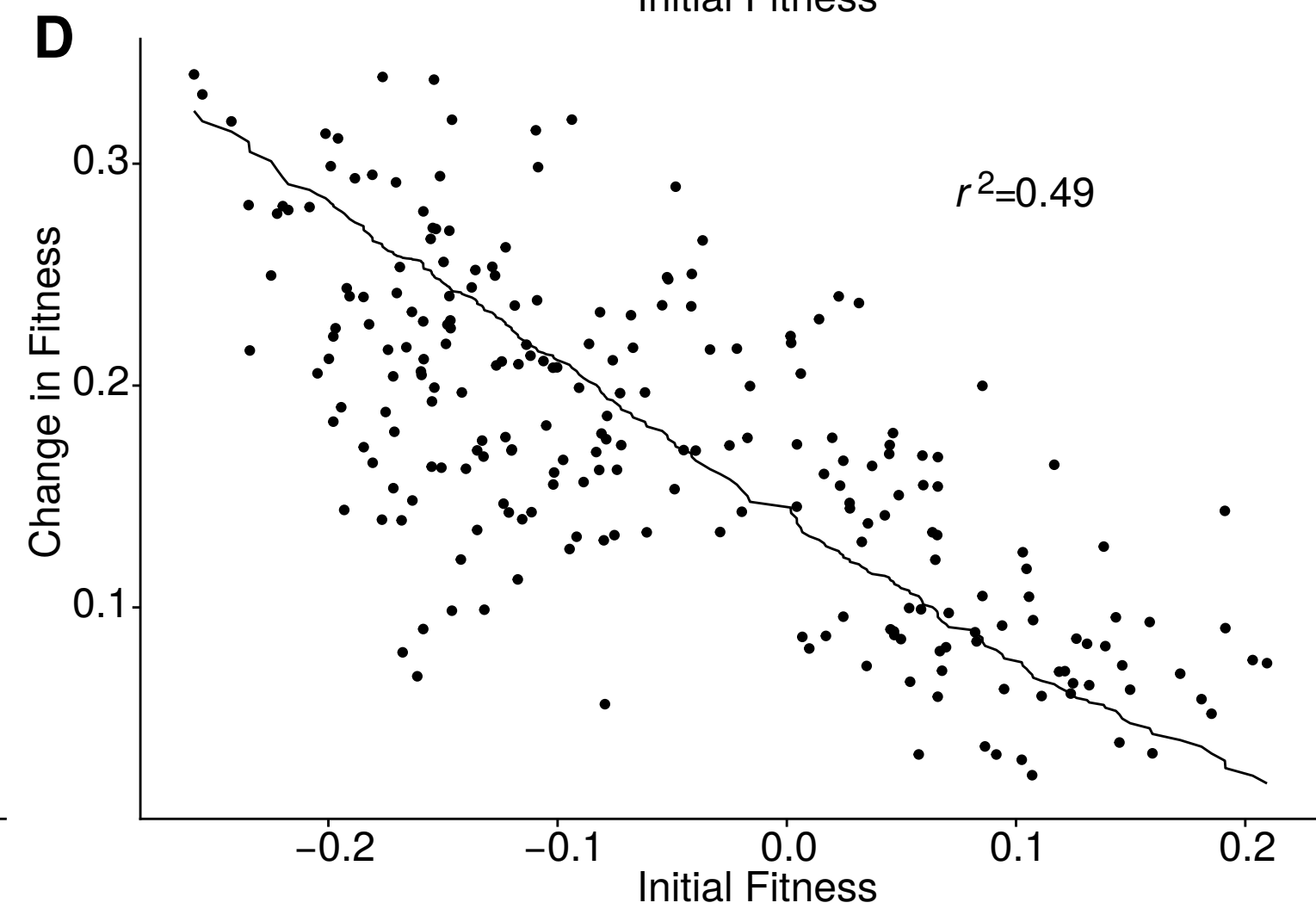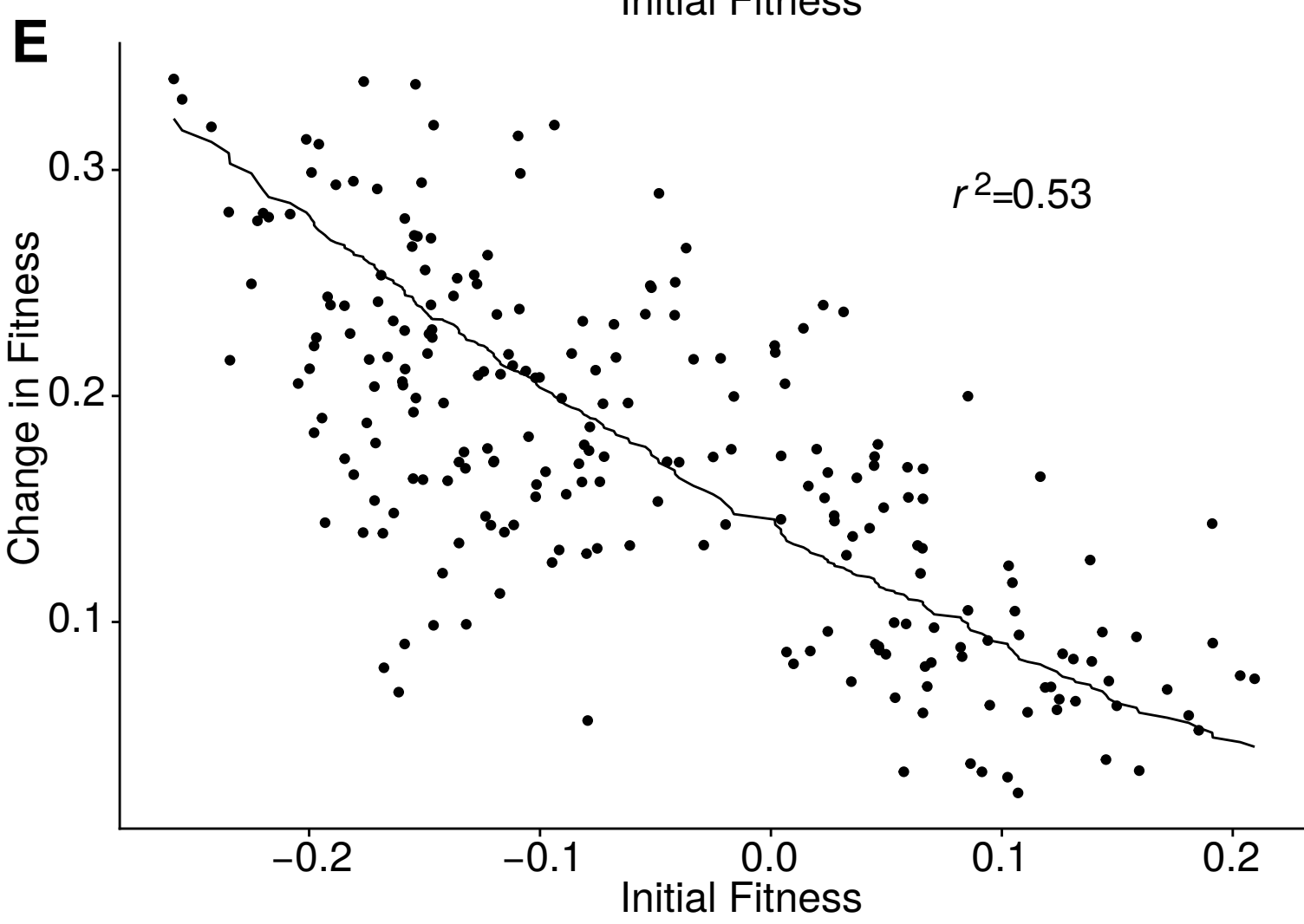

Supplement: Supplementary file 7 — Supplementary Figure 7 [file 41437_2018_143_MOESM7_ESM.pdf]

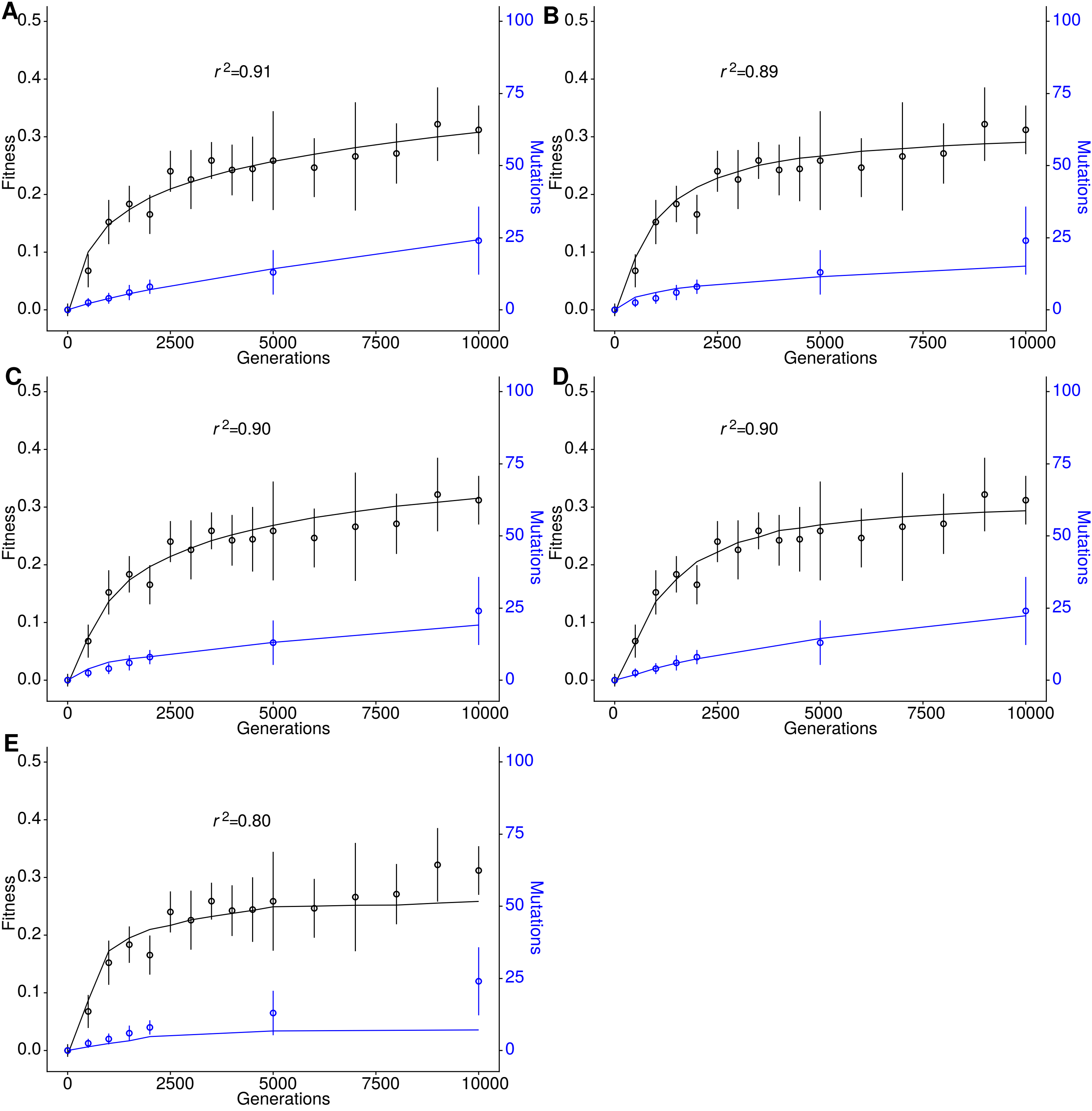

Supplement: Supplementary file 8 — Supplementary Figure 8 [file 41437_2018_143_MOESM8_ESM.pdf]

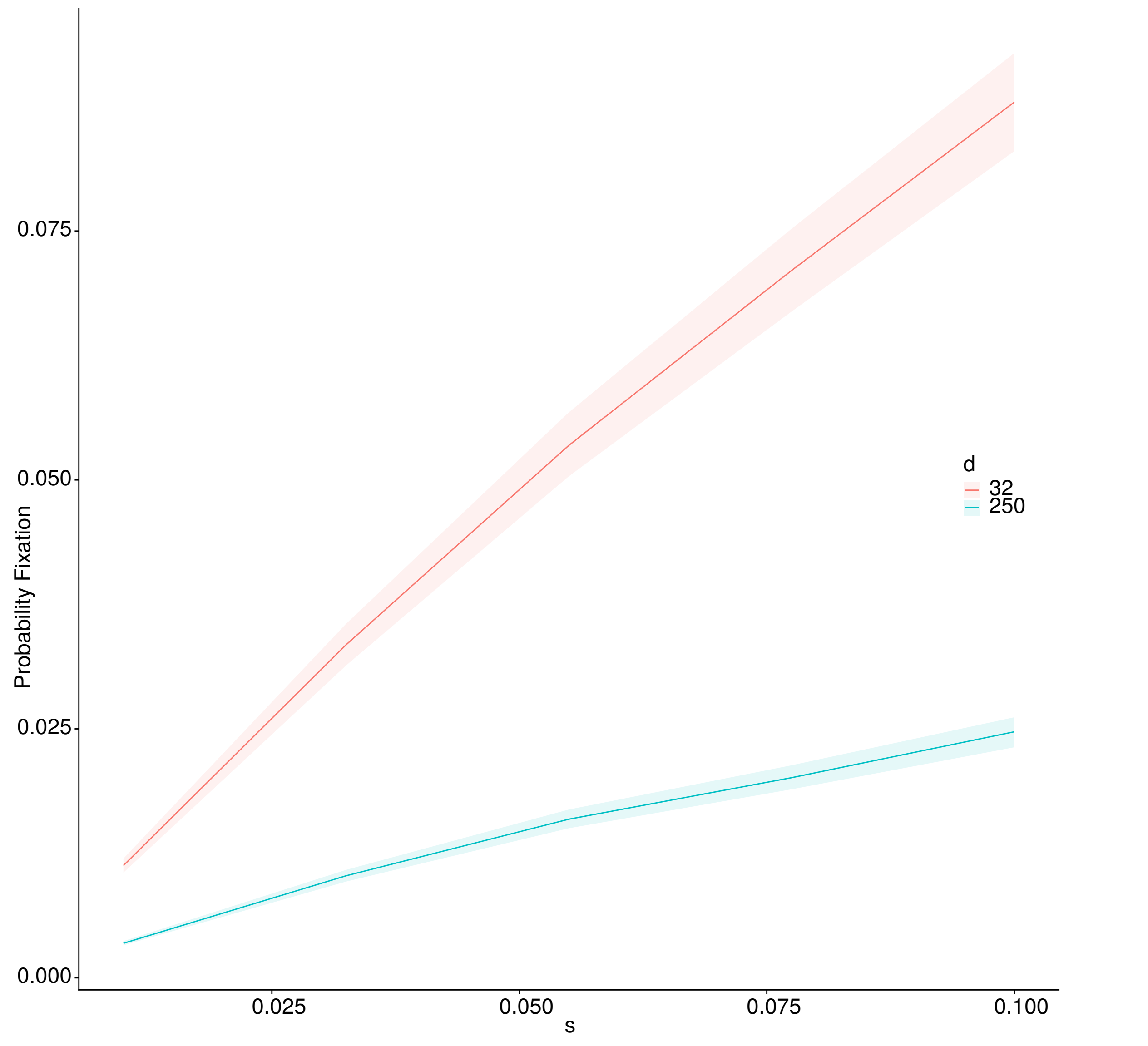

Supplement: Supplementary file 9 — Supplementary Figure 9 [file 41437_2018_143_MOESM9_ESM.pdf]
