# Supplementary material for: Power law fitness landscapes and their ability to predict fitness
Source: Heredity (Edinb). 2018 Sep 6;121(5):482–98. doi: 10.1038/s41437-018-0143-5 (PMC6180038; doi:10.1038/s41437-018-0143-5)
Supplement: Supplementary file 13 — Supplemental Tables [file 41437_2018_143_MOESM13_ESM.pdf]

Table S1: *Fission Yeast Genotypes*

Name	Genotype
Reference	arg1::padh1-loxP-kanMX6 leu1-32 ade6-M210 lys4::loxP-ura4-kanMX6 ura4+ matM:nat his1::mcherry hph
824	arg7::loxP-kanMX6 lys4::padh1-loxP-ura4-kanMX6 leu1-32 ura4-D18 ade6-M216 matM- natMX6 (invertion)
1058	arg1::loxP- kanMX6R arg7:: padh1-loxP- ura4+ - kanMX6R mat1-M::mat1-M-natMX6 leu1-32 ade6-M210 ura4-D18
1088	arg1::loxP- KanMX6R his1::padh1-loxP-ura4-kanMX6R mat1-M::mat1-M-natMX6 leu1-32 ade6-M210 ura4-D18
1089	his1::loxP- kanMX6R lys4::padh1-loxP- ura4+ - kanMX6R mat1-M::mat1-M-natMX6 leu1-32 ade6-M210 ura4-D18 matM
1279	his1::loxP- kanMX6R lys4::padh1-loxP-ura4- kanMX6R mat1-M::mat1-M-natMX6 leu1-32 ade6-M210 ura4+ matM
1563	arg7::padh1-loxP- kanMX6R lys4::loxP-ura4- kanMX6R mat1-M::mat1-M-natMX6 leu1-32 ade6-M216 ura4+
1565	arg1::padh1-loxP- kanMX6R arg7::loxP-ura4- kanMX6R mat1-M::mat1-M-natMX6 leu1-32 ade6-M210 ura4+ matM- natMX6R
1751	arg1::padh1-loxP- kanMX6R his1::loxP-ura4- kanMX6R mat1-M::mat1-M-natMX6 leu1-32 ade6-M210 ura4+ matM- natMX6R

Table S2: 95% *confidence interval in for Power Law parameters. Lower bound on top and upper bond on the bottom.*

Dataset	Rate Beneficial Mutations	Rate Neutral Mutations	α	k	λ
E. coli	$2.5e-05$	$9.5e-04$	0.28	0.11	15
	$9.5e-05$	$1.6e-03$	0.38	0.14	136
Budding yeast	$5.2e-05$	$1.9e-02$	0.39	0.15	1.1
	$6.8e-05$	$2.0e-02$	0.41	0.21	1.2
Budding yeast 30°	$1.6e-04$	$2.8e-04$	0.16	0.25	0.43
	$3.7e-04$	$5.2e-03$	0.34	0.30	11
Budding yeast 37°	$1.1e-04$	$1.4e-04$	0.93	0.38	0.92
	$3.0e-04$	$5.6e-03$	4.40	0.47	1.5
Fission yeast	$1.5e-06$		0.23	0.10	6.6
	$2.0e-04$		0.48	0.16	200

Table S3: *Best parameters for Stickbreaking model.*

Dataset	Rate Beneficial Mutations	Rate Neutral Mutation	λ	Maximum s
E. coli	$3.7e-05$	$1.1e-03$	0.024	0.32
Budding yeast	$1.0e-03$	$1.6e-02$	0.031	0.16
Budding yeast 30°	$3.2e-04$	$1.2e-03$	0.013	0.42
Budding yeast 37°	$3.3e-04$	$1.0e-03$	0.013	0.64
Fission yeast	$2.7e-06$		0.25	0.10

Table S4: *Best parameters for Saturation model.*

Dataset	Rate Beneficial Mutations	Rate Neutral Mutation	λ	Maximum s
E. coli	$5.2e-05$	$9.4e-04$	0.036	0.50
Budding yeast	$5.8e-05$	$1.9e-02$	0.14	0.28
Budding yeast 30°	$4.3e-04$	$7.6e-04$	0.02	0.57
Budding yeast 37°	$9.9e-05$	$8.2e-04$	0.022	0.91
Fission yeast	$6.5e-05$		0.51	0.15

Table S5: *Best parameters for Thermodynamics model.*

Dataset	Rate Beneficial Mutations	Rate Neutral Mutation	λ	Maximum s
E. coli	$1.7e-05$	$1.7e-03$	0.036	0.31
Budding yeast	$5.6e-07$	$2.e-02$	0.22	0.16
Budding yeast 30°	$2.1e-04$	$7.8e-02$	0.028	0.32
Budding yeast 37°	$1.8e-04$	$1.8e-03$	0.05	0.32
Fission yeast	$4.4e-05$		0.12	0.13

Table S6: *Best parameters for FGM model.*

Dataset	Rate Mutations	Maximum s	σ	Dimensions	Q
E. coli	$8.6e-04$	0.35	5.6	5	2.2
Budding yeast	$1.4e-02$	0.2	0.024	89	2.9
Budding yeast 30°	$6.6e-04$	0.25	0.017	72	2.4
Budding yeast 37°	$1.4e-04$	0.43	0.01	25	4.7
Fission yeast	$1.1e-04$	0.096	0.026	29	7.1

Table S7: *Goodness of Fit p-values*

Model	Fission Yeast	Budding yeast 30°	Budding yeast 37°	Budding yeast	*E. coli*
Power Law	0.551	0.612	0.984	0.680	0.984
Stickbreaking	0.050	0.520	0.330	0.994	0.984
Saturation	0.646	0.646	0.072	0.984	0.994
Thermodynamics	0.646	0.646	0.161	0.984	0.984
FGM	0.646	0.671	0.747	0.357	0.646
